# Insecticidal activity of essential oils from American native plants against *Aedes aegypti* (Diptera: Culicidae): an introduction to their possible mechanism of action

**DOI:** 10.1038/s41598-023-30046-8

**Published:** 2023-02-20

**Authors:** Jonny E. Duque, Diana L. Urbina, Luis C. Vesga, Luis A. Ortiz-Rodríguez, Thomas S. Vanegas, Elena E. Stashenko, Stelia C. Mendez-Sanchez

**Affiliations:** 1 Centro de Investigaciones en Enfermedades Tropicales – Cintrop, Departamento de Ciencias Básicas, Escuela de Medicina, Universidad Industrial de Santander, Parque Tecnológico y de Investigaciones Guatiguará Km 2 El Refugio, Piedecuesta, Colombia; 2 Grupo de Investigación en Bioquímica y Microbiología (GIBIM), Escuela de Química, Universidad Industrial de Santander, Bucaramanga, Colombia; 3 Centro de Investigación en Biomoléculas – CIBIMOL y Centro Nacional de Investigación para la Agroindustrialización de Plantas Aromáticas y Medicinales Tropicales – CENIVAM, Universidad Industrial de Santander, Bucaramanga, Colombia

**Keywords:** Biochemistry, Cheminformatics

## Abstract

Searching for new bioactive molecules to design insecticides is a complex process since pesticides should be highly selective, active against the vector, and bio-safe for humans. Aiming to find natural compounds for mosquito control, we evaluated the insecticidal activity of essential oils (EOs) from 20 American native plants against *Aedes aegypti* larvae using bioassay, biochemical, and in silico analyses. The highest larvicide activity was exhibited by EOs from *Steiractinia aspera* (LC_50_ = 42.4 µg/mL), *Turnera diffusa* (LC_50_ = 70.9 µg/mL), *Piper aduncum* (LC_50_ = 55.8 µg/mL), *Lippia origanoides* (chemotype thymol/carvacrol) (LC_50_ = 61.9 µg/mL), *L. origanoides* (chemotype carvacrol/thymol) (LC_50_ = 59.8 µg/mL), *Hyptis dilatata* (LC_50_ = 61.1 µg/mL), *Elaphandra quinquenervis* (LC_50_ = 61.1 µg/mL), and *Calycolpus moritzianus* (LC_50_ = 73.29 µg/mL) after 24 h. This biological activity may be related to the disruption of the electron transport chain through the mitochondrial protein complexes. We hypothesized that the observed EOs' effect is due to their major components, where computational approaches such as homology modeling and molecular docking may suggest the possible binding pose of secondary metabolites that inhibit the mitochondrial enzymes and acetylcholinesterase activity (AChE). Our results provided insights into the possible mechanism of action of EOs and their major compounds for new insecticide designs targeting the mitochondria and AChE activity in *A. aegypti* for effective and safe insecticide.

## Introduction

The new way to design green insecticides includes plant extracts (PE) and essential oils (EOs), focusing on environmentally eco-friendly products^1–3^. Therefore, it is crucial to begin pesticide design with a selective screening to know if the product is suitable for insect control and then to continue its refinement processes to support a future commercial insecticidal formulation. In this sense, and due to the necessity of discovering new products for mosquito control that are environmentally safe and fit into a philosophy of sustainability from a safety perspective, further findings on bioinsecticides must bring information about the toxicity of their components^3–5^.

Fortunately, there is extensive documentation about plants with insecticidal activity against *A. aegypti*, even with comprehensive details on their chemical characterization to make them commercial biopesticides^6–8^. Within this extensive documentation, some studies highlight features that should have a botanical insecticide against mosquito larvae. In the first instance, to acquire this connotation, the lethal concentration (LC) LC_50_ of EO or PE should be less than 100 ppm, also considering their physicochemical properties^9^. Classification for the mechanism of action of insecticide substances has also been described and includes protein denaturalization, enzymatic inhibition, and membrane disintegration^7^.

When searching for components to design green insecticides, it is crucial to have a primary approximation of their compounds and how they contribute to the synergy of the biocidal effect. For this, computational approaches have become pivotal tools for bioactive compound identification for insecticide formulation^10^. Structure-based virtual screening (SBVS) is probably the most widely used in the lead compound identification in biological assays; however, this computational approach has some limitations related to the compounds library quality that has an important effect on SBVS results as well as on the use of the rule-of-five including molecular weight (MW), LogP, H-donors, H-acceptors, and different physicochemical filters for the library^11^. Additionally, scoring function and the prediction of correct binding poses with high accuracy is still challenging; thus, docking scores have little confidence in ranking potential ligands^12,13^. However, despite these limitations, structure-based virtual screening, molecular docking simulations, and different computational approaches have been successfully used to identify potential leads for new drug development^14,15^. In this sense, the SBVS and other computational tools have been used recently to identify bioactive molecules aiming to inhibit specific molecular targets such as the nicotinic acetylcholine receptor and its use in insecticidal formulation^10,16^.

In this work, we showed that not only should the new EOs or PE's insecticide action be considered, but their components must be included in silico analyses to know their ADME (absorption, distribution, metabolism, and excretion) properties and possible human health risks. Toxicity also is a parameter that can be explored when chemical characterization of natural biocide is carried out^17^. Using computational analysis, the possible mechanism of action could be predicted for each essential oil or plant extract according to their components, which will significantly help to support its insecticide activity when in vivo experiments are validated^3,18^. This work showed the larvicidal activity of EOs from American native plants. Besides, based on their potential insecticide activity, we evaluated the EO’s effect and its possible mechanism of action on mitochondria and acetylcholinesterase as main targets to design new bio-products for mosquito control.

## Material and methods

### The essential oil obtaining and characterization

All botanical specimens used to screen the larvicidal activity were collected from different Colombian places under permission from the Ministerio de Ambiente y Desarrollo Sostenible (MADS) through its Dirección de Bosques, Biodiversidad y Servicios Ecosistémicos and access to genetic resources and derived products for the program the Unión Temporal Bio-Red-CO-CENIVAM (Resolution 4 June 0812 2014). Authorization for collecting wild species specimens from biological diversity for non-commercial scientific research purposes granted by the National Environmental Licensing Authority—ANLA (Resolutions 004 22 of January 2015 and 11 March 0260 2016). The material was identified up to the species level and was deposited in the National Herbarium. This approach complies with local and national regulations. The extraction process was made by the “Centro de Investigación en Biomoléculas—CIBIMOL y Centro Nacional de Investigación para la Agroindustrialización de Plantas Aromáticas y Medicinales Tropicales—CENIVAM”. To obtain the EO’s and their characterization following our previous methodology^19–22^. Twenty different American native plants’ EO’s were obtained by microwave-assisted hydrodistillation. Each essential oil (50 mg) was dissolved in 1 mL of CH2Cl2, and an aliquot of this dilution (2 µL) was injected into a gas chromatograph coupled to a mass selective detector.

Analysis was performed on a gas chromatograph, GC 6890 Plus (Agilent Technologies, AT, Palo Alto, CA, USA), equipped with a mass selective detector MS 5973 Network (AT, Palo Alto, CA, USA). USA), using electron ionization (EI, 70 eV). Helium (99.995%, AP gas, Messer, Bogotá, Colombia) was used as a carrier gas, with an initial inlet pressure at the head of the column of 113.5 kPa; the carrier gas volumetric flow rate during the chromatographic run was held constant (1 mL/min). The injection mode was split (30:1), and the injector temperature was maintained at 250 °C. Compound separation was carried out in two capillary columns, one with the polar stationary phase of poly(ethylene glycol), PEG (DB-WAX, J & W Scientific, Folsom, CA, USA) of 60 m × 0.25 mm (i.d.) × 0.25 μm and another nonpolar column with 5%-phenyl-poly(methylsiloxane), 5%-Ph-PDMS (DB-5MS, J & W Scientific, Folsom, CA, USA) of the same dimensions. In the polar column (DB-WAX), the oven temperature was programmed from 50 °C (5 min) to 150 °C (7 min), at 4 °C/min, and then up to 230 °C (50 min), at 4 °C/min. In the non-polar column (DB-5MS), the temperature of the chromatographic oven was programmed from 45 °C (5 min) to 150 °C (2 min), at 4 °C/min, then up to 300 °C (10 min), at 5 °C/min. The GC/MS transfer line temperature was set at 230 °C when the polar column was used and at 300 °C for the non-polar column. The ionization chamber and the quadrupole temperatures were 250 °C and 150 °C, respectively. The mass range for the acquisition of ionic currents was m/z 45–450 u, with an acquisition speed of 3.58 scan/s. Data were processed with MSDChemStation G1701DA software (AT, Palo Alto, CA, USA). The integration parameters were the following: threshold = 18 and "rejection area" of the peak over the baseline less than 1%. The identification of compounds was carried out based on their linear retention indices (LRI), calculated from the retention times of the compound of interest and the C6-C25 and C8-C40 n-alkanes (Sigma-Aldrich, San Luis, MO, USA), according to Eq. (1),

1 $${\text{LRI }} = \, \left( {{1}00 \, \times {\text{ n}}} \right) \, + {1}00 \, \times \left[ {{\text{tRx }} - {\text{ tRntRN}} - {\text{ tRn}}} \right]$$

LRI = Linear retention index of the compound of interest

n and N = Number of carbon atoms of n-alkanes, eluting before (n) or after (N) the compound of interest (x)

tRX = Retention time of the compound of interest (min)

tRN and tRn = Retention times (min) of n-alkanes eluting before (n) or after (N) the compound of interest (x)

For tentative identification, experimental mass spectra were compared to those from Adams (2007), NIST (2017), and Wiley (2008) spectral databases. Only spectral matches above 95% were accepted. Some compounds were confirmed by comparing their LRI and their mass spectra with the available standard substances (S. Table 1, S. Figs. 1–20).

### Biological material

The larvicidal activity was performed using *A. aegypti* mosquitoes from the Rockefeller line. The insects were kept inside breeding cages (40 × 40 × 40 cm) under insectary conditions like humidity (70 ± 5%), photoperiod (12:12), and temperature (27 ± 5 °C). The *A. aegypti* colonies were fed with a 10% honey sugar solution. *A. aegypti's* eggs were collected and placed in chlorine-free water containers to induce hatching. Subsequently, the female mosquitoes were offered blood from a Wistar rat bred at the Universidad Industrial de Santander's vivarium^21,22^. Complying with the laboratory animal handling procedures and under the approval of the Ethics Committee “*Comité de ética en investigación científica CEINCI*”: Scientific Research Ethics Committee of the Industrial University of Santander (acronym in Spanish CEINCI); Minutes No. 22, Dec 6, 2019). Furthermore, the study was carried out in compliance with the arrive guidelines and following the 1989 Colombian Law 84 (Chapter IV, Art. 23–26) and Resolution 8430 (1993, Title IV, Art. 83–93) that regulates animal research in Colombia.

### Larvicidal activity

The insecticidal activity of each EO was evaluated according to the method described by the WHO^23^. Although, we also used our insecticidal activity protocols to screen the EOs with higher activity broadly^9,21,22^. Each EO was screened at an exploratory concentration (EC) of 100 µg/mL in larvae between the third and fourth instars. Then, the EOs with higher mortality than 75% were selected as promissory insecticides; the control test used dimethyl sulfoxide (DMSO, 0.5%) and mineral water. Additionally, temephos was used as a positive control at 0.032 µg/mL. Mortality rates and lethal concentrations LC50 and LC95 were determined by mortality and survival bioassays subjected to Probit analysis (Finney, 1971). Only the lethal concentrations (LC_50_ and LC_95_) were determined for those EOs that overcame the minimum mortality criteria (EC with LC_50_ under 100 µg/mL). Each bioassay was performed by four independent experiments (N = 120 larvae by treatment) and was replicated in triplicate. The EOs with the higher larvicidal activity was further evaluated in mitochondria and acetylcholinesterase.

### Larvae homogenization

To obtain total proteins for the enzymatic tests, we used one hundred *A. aegypti* larvae at instar L3-L4, the larvae were suspended in an isotonic medium described by Castillo *(2019)* (250 mM sucrose, 10 mM HEPES, 1 mM EGTA), and homogenized with a tissue grinder Potter–Elvehjem. The homogenate was filtered through a glass wool syringe to remove large exoskeleton residues. The final product was quantified for total protein by the Bradford method^24^, using BSA as the standard.

### Essential oil effect on mitochondrial enzymatic activity

The EO effect on electron transport through mitochondrial complexes was evaluated polarographically according to Singer^25^ using a high-resolution respirometry Oroboros-instrument oxygraph O2K. The reaction medium contained phosphate buffer solution (80 mM) pH 7.4, EDTA (50 µM), and homogenated larvae (0.1 mg/mL). The selected EOs were incubated for 2 min at LC_50_ before the reaction started, and NADH (0.2 mM) oxidation rate to NAD^+^ and succinate (10 mM) oxidation rate to fumarate were expressed by the oxygen consumption ratio.

### Acetylcholinesterase inhibition assay

The EO’s effect on AChE activity was evaluated using the method described by Ellman (1961)^26^. The colorimetric assay measures the formation rate of thiocholine by the hydrolysis of acetylthiocholine. The thiocholine generated in situ reacts with Ellman's reagent DTNB (5,5′-dithiobis(2-nitrobenzoic acid), and the chromophore formed has an absorbance maximum at 410 nm. LC_50_ of each EO was evaluated on 0.1 mg/mL of the L3-L4 larvae homogenized as described in the previous section, and commercial acetylcholinesterase from *Electrophorus electricus* was used as a biological model. Three independent tests with triplicate were carried out on the homogenized larvae and the commercial AChE. For the experiment, buffer PBS, EO, and the enzyme or homogenized larva were added to a 96-well plate with a final volume of 200 μL. It was incubated with gentle agitation for 10 min, and the Ellman reagent was added to initiate the reaction while it was monitored with a Thermo Scientific™ Multiskan™ FC Microplate Photometer. The results were recorded as the maximum reaction rate.

### The essential oil effect on cell viability

The MTT (3-(4,5-dimethylthiazol-2-yl)-2,5-diphenyltetrazolium bromide) assay was performed on cell line VERO to verify the EO’s cytotoxic effect. VERO cells were grown in EMEM medium, supplemented with 7% fetal bovine serum and 100 µg/mL gentamicin (pH 7.4). The cell culture was maintained at 37 °C and 5% CO_2_. Cell viability was determined by the method described by Mosmann^27^. With this method, viable and metabolically active cells reduce the tetrazolium salt (MTT) to formazan crystals, which are soluble in DMSO, and the absorbance is measured at 570 nm. For this, cells were plated in 96-well plates (7500 cells/well) and incubated for 24 h, as described before. Then, cells were treated with CL_50_ EOs for 48 h. Briefly, MTT solution (0.5 mg/mL) was used and incubated for 3 h at 37 °C, the formazan crystals were dissolved with DMSO, and the absorbance was measured. Cell viability values were expressed as a percentage of control (without essential oil).

### Computational approaches

Since the insecticidal activity of EOs could be related to the effect of their secondary metabolites on targets such as mitochondria and acetylcholinesterase, previously reported as main targets for insecticide design^3,28^. We used computational approaches such as the homology model and molecular docking to explore these potential targets for achieving potent and selective inhibitors.

### Calculation of ADMET properties

A set of ADMET properties for our 57 selected compounds was calculated using the QikProp program in Schrödinger suite 2022-1^29^. QikProp generates physically relevant descriptors and uses them to perform ADMET predictions. Briefly, the ADME-compliance score—drug-likeness parameter (indicated by #stars) was used to assess our selected compounds’ pharmacokinetics profiles.

### Homology modelling

Crystal structures of mitochondrial complexes for *A. aegypti* are not available in the Protein data bank (PDB). Therefore, the FASTA sequences of mitochondrial complexes I, II, III, and IV were obtained from the UniprotKB^30^. Subunit 5 from complex I (ID: B0FWD3), subunit A from complex II (ID: A0A6I8TJS2), and Cyt B from complex III (ID: B0FWD7) were selected. The suitable templates for homology modeling were identified on PDB using PSI-BLAST with the non-redundant PDB database, and models were built using Prime in Schrödinger suite 2022-1^31,32^. Then, target and template sequences were aligned using the Clustal W method in Prime, followed by manual adjustment to avoid gaps. Further, the prime refinement protocol was used to predict the side chains and minimized using OPLS3e force field^33^, and validation of the models was done using tools such as Ramachandran Plot.

### Protein preparation and molecular docking

The protein preparation was performed using the Schrödinger Drug Discovery suite for molecular modeling (version 2020-4). The crystal structure of acetylcholinesterase (PDBID: 1QON, resolution 2.72 Å^34^) was obtained from Protein Data Bank (PDB, www.rcsb.org) and was prepared using Protein preparation wizard tool^35^ to fix the protonated states of amino acids residues, adding polar hydrogens and missing side-chain atoms. Besides, a missing loop between amino acids Lys 102-Gln 136 was generated and optimized using Prime^31^.

All secondary plant metabolites, as well as the acetylcholinesterase co-crystalized ligand (9-(3-iodobenzylamino)-1,2,3,4-tetrahydroacridine, herein referred as I40) were drawn using software Maestro and were prepared using LigPrep^36^ to generate the three-dimensional conformation, calculate the partial atomic charges, and to adjust the protonation states at pH 7.4 using the force field OPLS3e^33^. Molecular docking studies were AutoDock vina, using the Lamarckian genetic algorithm (LGA) and the Solis & Wets local search method. Ligands were docked in a grid box of 15 Å as follows: for subunit 5 from complex I, Sitemap tool^37,38^ was employed to identify the binding site, while the Grid box was centered in residues Thr 302, His 290, Glu 303, and Gly 99 for subunit A in complex II; residues Phe 275, Tyr 274, and Tyr 132 for Cyt B in complex III. Finally, for acetylcholinesterase crystal structure, ligands were docked in the same place as co-crystalized ligands I40. Docking poses were selected by visual inspection based on their common interactions with relevant residues for each target.

### Statistical analysis

The mortality effect was evaluated at 24 h and 48 h treatment to each EO concentration used in this work, and the survival larval were subjected to Probit analysis^39^. Cell viability values were expressed as a percentage of control, and for all assays, ANOVA and Tukey test was applied to data with normal distribution, and treatments were considered statistically significant with* p* ≤ 0.05*.* The results of the enzymatic activity experiment were analyzed in Prism GraphPad 8 software.

## Results

### Essential oils distillation and identification of their major components

The EOs were distilled from American native plants obtained by microwave-assisted hydrodistillation (MWHD), exhibiting different extraction yields. The major metabolites in each EO are reported in S. Table 2.

### EOs from American plants as a potential insecticide against *A. aegypti*

**Figure 1:**
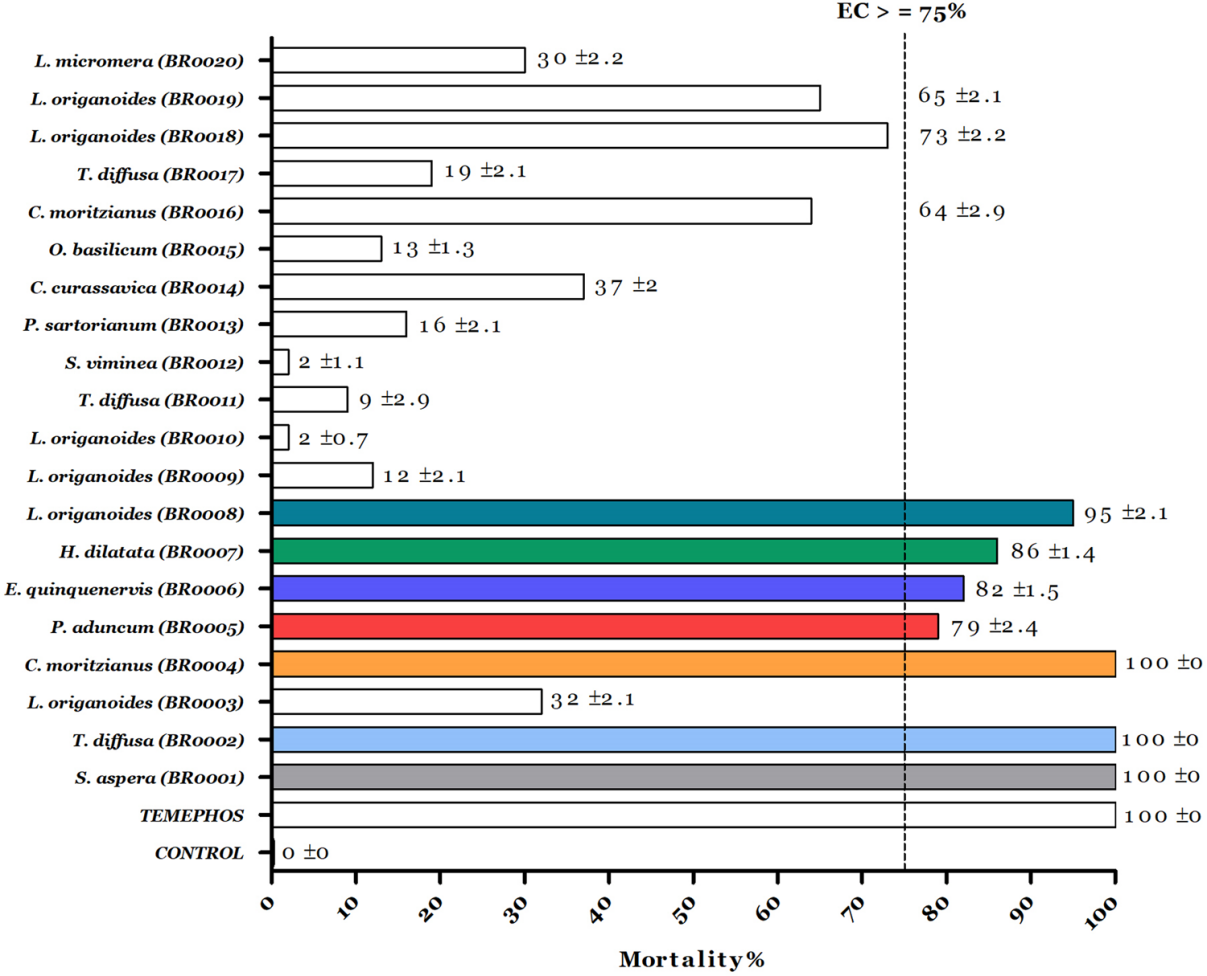
The essential oils mortality rate at the exploratory concentrations (EC) 100 µg/mL. The dashed line represents the activity threshold considered a promissory larvicide. Colored bars represent the promissory EOs as larvicide; temephos was used as a positive control with an LC_90_ of 0.032 µg/mL.

After the EOs were chemically characterized, their mortality rates in *A. aegypti* larvae were evaluated to know their potential use as a possible insecticide. As shown in Fig. 1, seven of the twenty evaluated EO showed a high mortality rate at the exploratory concentration (EC) of 100 ppm. In this sense, the essential oils from *S. aspera* (100% ± 0.0), *T. diffusa* (100% ± 0.0), *P. aduncum* (79% ± 2.4), *L. origanoides *(chemotype thymol/carvacrol) (73% ± 2.2), *L. origanoides* (chemotype carvacrol) (95% ± 2.1), *H. dilatata* (86% ± 1.4), *E. quinquenervis* (82% ± 1.5), and *C. moritzianus* (100 ± 0), showed the higher activity with a mortality rate over 75% at EC.

Subsequently, the EOs with the higher activity were tested at multiple concentrations to obtain the LC_50_ and LC_95_ values. As shown in Table 1, within the seven selected EOs tested, *S. aspera* showed the best larvicide activity with an LC_50_ of 42.4 and 90.5 µg/mL at 24 and 48 h, respectively. All EOs studied showed an insecticide activity with LC_50_ lower than 100 µg/mL at 24 h.

**Table 1:** Essential oil lethal concentrations on *A. aegypti* larvae.

EO code	24 h			48 h		
	LC_50_	LC_95_	x^2^	LC_50_	LC_95_	x^2^
*S. aspera* (001)	42.4 (33.4–50.7)	90.5 (71.1–151.2)	5.1	42.2 (28.33–54.71)	89.6 (65.85–234.14)	4.6
*T. diffusa* (002)	70.9 (67.00–75.09)	97.9 (89.86–112.49)	8.2	*58.5 (54.84–62.31)*	82.4 (75.35–94.88)	9.1
*C. moritzianus* (004)	55.8 (43.57–76.50)	83.0 (65.28–232.36)	5.2	55.8 (43.45–73.95)	87.8 (68.27–204.87)	5.9
*P. aduncum* (005)	61.9 (58.95–65.13)	102.0 (93.28–114.76)	2.2	57.3 (52.53–62.17)	94.7 (84.82–110.62)	5.4
*E. quinquenervis* (006)	59.8 (51.44–71.30)	116.1 (91.23–190.97)	7.1	48.8 (43.87–53.71)	143.4 (120.17–184.06)	1.9
*H. dilatata* (007)	61.1 (54.27–69.88)	96.6 (80.77–147.52)	8.0	56.7 (55.76–61.89)	89.4 (79.36–110.47)	4.6
*L. origanoides *(Chemotype carvacrol + *p*-cymene) (008)	73,29 (63.51–92.10)	112,13 (90.01–199.15)	14.5	75.4 (62.77–92.61)	126.2 (100.02–247.02)	4.0

Values are expressed in µg/mL at 24 h and 48 h.

Lethal concentration 50 (LC_50_), Lethal concentration 90 (LC_90_). x^2^, indicates dependence between LC_50_ and LC_90_.

### Electron transport through the mitochondrial complexes is inhibited by essential oils

**Figure 2:**
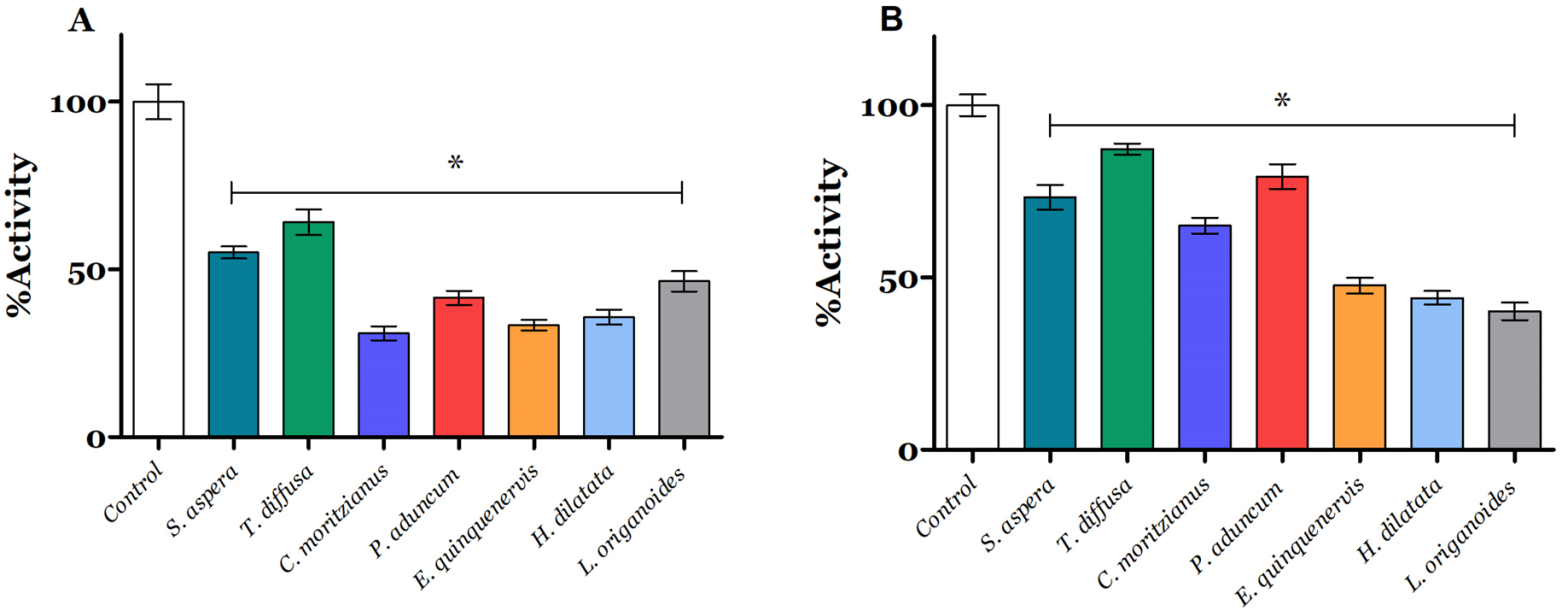
Effect of EOs on mitochondrial electron transport through the mitochondrial respiratory chain. (**A**) NADH oxidase activity; (**B**) Succinate oxidase activity. 100% activity represents 686.17 pmolO_2_/s*mg total protein and 620.88 pmolO_2_/s*mg total protein for NADH and succinate oxidase activity, respectively. Each value represents the mean ± SD of three independent experiments. *Significant statistical differences with respect to control *p* < 0.05.

With the seven EOs selected that showed good larvicidal activity, the determination of possible mechanisms of their action was continued, initially, on electron transport through the mitochondrial respiratory chain. We evaluated LC_50_ of each EO on larvae homogenate by measuring the oxygen consumption rates after NADH and succinate addition as oxidable substrates. As shown in Fig. 2A, all tested EOs decreased the oxygen consumption rate with statistically significant differences when NADH was used as an oxidable substrate, *S. aspera* reduced the activity by 44.9%; *T. difussa* 35.9%, *C. moritzianus* 69%*, P. aduncun* 41.6%*, E. quinquenervis* 66.7%*, H. dilatata* 64.2%*, and L. origanoides* reduces the activity by 53.5%*.* Similarly, EOs decrease the succinate oxidase activity on *A. aegypti*. As shown in Fig. 2B, *S. aspera* decreased the succinate oxidase up to 26.7%; *T. difussa* 12.7%, *C. moritzianus* 34.9%*, P. aduncum* 20.6%*, E. quinquenervis* 52.3%, *H. dilatata* 55.9%, and *L. origanoides* 59.8%.

### Acetylcholinesterase activity is inhibited by essential oil action

**Figure 3:**
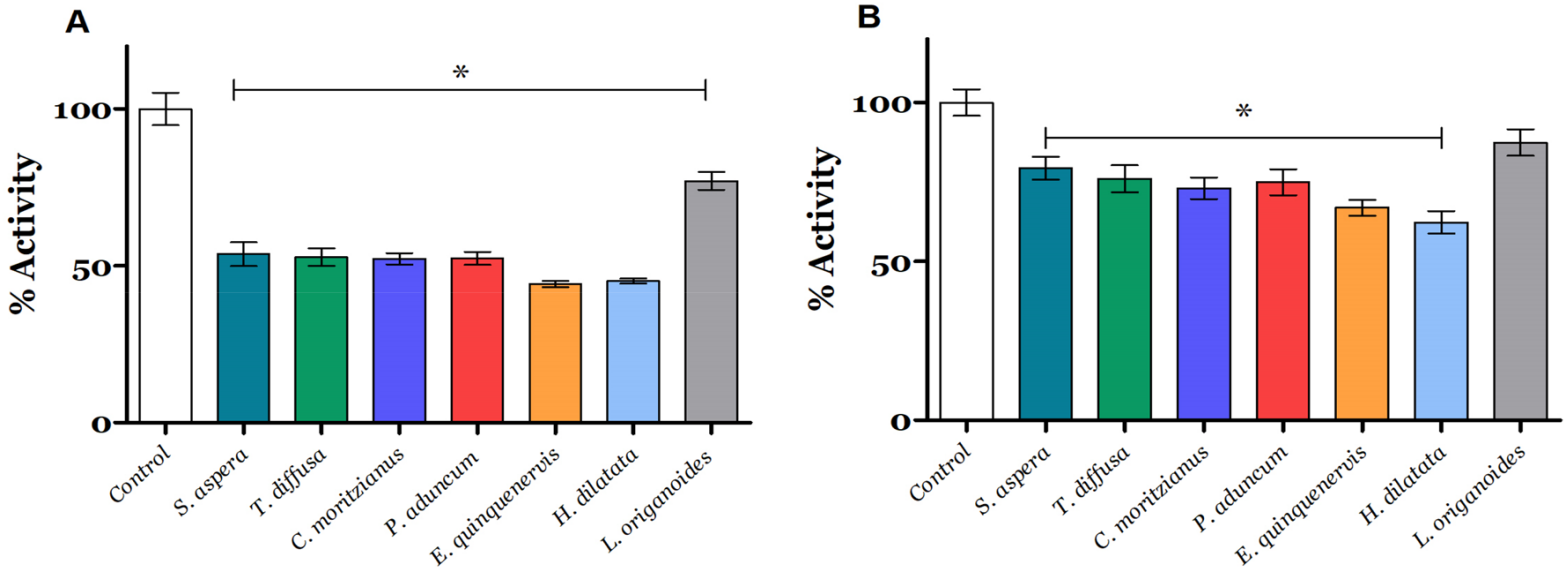
Effect of EOs on acetylcholinesterase activity. (**A**) *A. aegypti* larvae; (**B**) commercial Acetylcholinesterase from *Electrophorus electricus.* All values were normalized with respect to the negative control value representing 100% activity with 3.37 nmol of reduced DTNB/min^−1^ mg^−1^ of total protein and 9.33 nmol of reduced DTNB/min^−1^ mg^−1^ of total protein (0.1 mg), respectively. Each value represents the mean ± SD of three independent experiments. ANOVA and Tukey post hoc tests were employed. *Represent statistically significant with *p* ≤ 0.05.

Acetylcholinesterase is one of the main cholinesterases in the body involved in synaptic transmission. We evaluated the effect of each EO at LC_50_ on AChE activity. As shown in Fig. 3A, all EOs reduced the acetylcholinesterase activity in *A. aegypti* as follows: *S. aspera* by 46.2%; *T. difussa* 47.2%, *C. moritzianus* 47.7%*, P. aduncum* 47.6%*, E. quinquenervis* 55.7%*, H. dilatata* 54.8%*, and L. origanoides* reduced the AChE activity by 22.9%*.*

Additionally, the effect of EO in a commercial AChE activity from *Electrophorus electricus* was also evaluated at LC_50,_ aiming to compare the effect of each EO on purified AChE. As shown in Fig. 3B,E*. quinquenervis* and *H. dilatata* had the highest inhibition by reducing the AChE activity by 33% and 37.7%, respectively. Interestingly, the EO effect on commercial AChE activity was similar to the AChE on *A. aegypti* where *S. aspera* decreased the AChE activity by 20.6%, *T. diffusa* 23.9%, *C. moritzianus* 26.9%, *P. aduncum* 25%, and *L. origanoides* (chemotype carvacrol) reduced the AChE activity by 12.6%.

### Essential oil effect on cell viability

**Figure 4:**
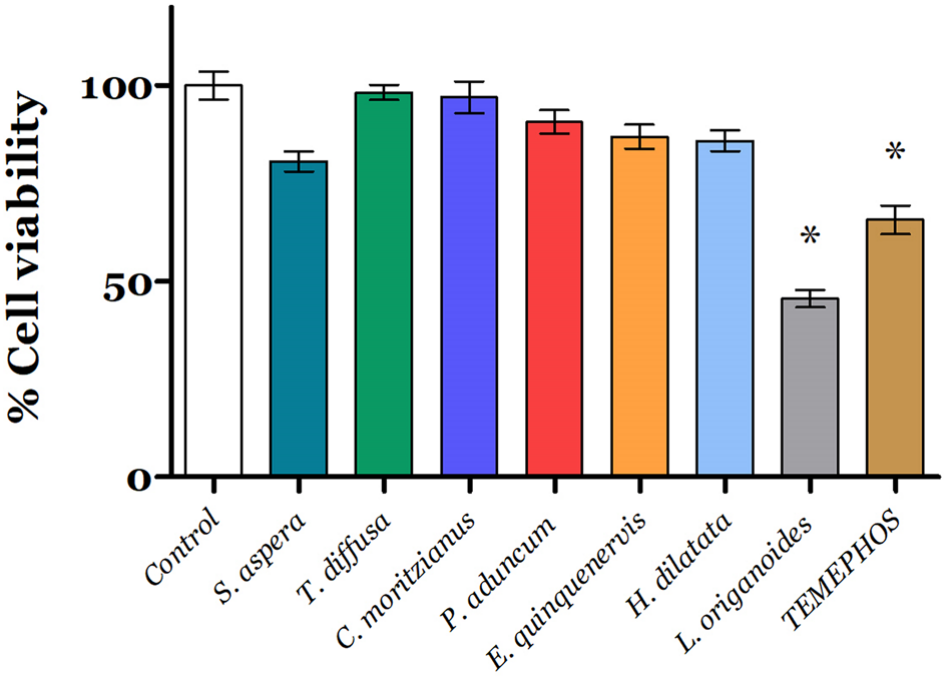
Cytotoxic effect of essential oils on Vero cells. Each value represents the mean ± SD of three independent experiments. *Significant statistical differences to the control *p* < 0.05; temephos was used as a positive control.

The components included as a good insecticide should not be toxic to other species; thus, we evaluated the effect of EO with the higher insecticidal activity on Vero cells viability. As shown in Fig. 4, all EOs were tested at their LC_50_, and after 48 h treatment, *L. origanoides* oil had a cytotoxic effect by decreasing up to 45.59 ± 6.8 the cell viability on Vero cells.

### Plant secondary metabolites bind on relevant targets for insecticide activity

One of the principal aims of this work was to explore the possible binding mode of selected plant secondary metabolites on relevant targets, trying to explain the observed insecticide activity. For this, some subunits of mitochondrial complexes I, II, III, and IV and acetylcholinesterase, were selected as the main targets due to their relevant role in mosquitoes' bioenergetics and synapsis^2,40,41^. For this, we first evaluated the ADME properties of these metabolites, finding no violation of rule-of-five and Jorgensen rule as shown in S. Table 3; besides, our docking results suggest that secondary metabolites may inhibit the selected *Aedes*’ mitochondrial targets as well as acetylcholinesterase (Table 2).

**Table 2:** Docking score and free binding energy calculation of best-ranked plant metabolites against selected molecular targets.

Target	Compound	Docking score (kcal/mol)
Complex I	*trans*-β-caryophyllene	− 5.8
	Viridiflorol	− 5.5
	Germacrene D	− 5.3
	Carvacrol	− 4.9
	*γ*-terpinene	− 4.7
	*p*-cymene	− 4.7
	Thymol	− 4.7
	*β*-pinene	− 4.6
	Limonene	− 4.6
	Sabinene	− 4.6
Complex II	Thymol	− 6.2
	Carvacrol	− 5.8
	Viridiflorol	− 5.7
	Limonene	− 5.4
	*γ*-terpinene	− 5.3
	*p*-cymene	− 5.3
	Sabinene	− 5.0
	*β*-pinene	− 4.9
	*trans*-β-caryophyllene	− 4.6
	α-pinene	− 4.5
Complex III	*p*-cymene	− 6.6
	Sabinene	− 6.6
	Carvacrol	− 6.4
	*γ*-terpinene	− 6.4
	Limonene	− 6.4
	α-pinene	− 6.0
	*delta*-carene	− 6.0
	Thymol	− 5.8
	β-pinene	− 5.4
	*trans*-β-caryophyllene	− 4.7
Complex IV	Viridiflorol	− 4.0
	Carvacrol	− 3.9
	*trans-*β-caryophyllene	− 3.9
	Germacrene D	− 3.8
	*γ*-terpinene	− 3.7
	*p*-Cymene	− 3.6
	Sabinene	− 3.6
	Thymol	− 3.6
	*delta*-carene	− 3.4
	Limonene	− 3.4
Acetylcholinesterase	*p*-Cymene	− 6.9
	Carvacrol	− 6.8
	*γ*-terpinene	− 6.8
	*delta*-carene	− 6.6
	Limonene	− 6.6
	Vidiriflorol	− 6.5
	*trans*-β-caryophyllene	− 6.5
	Germacrene D	− 6.4
	β-pinene	− 6.4
	Sabinene	− 6.3

**Figure 5:**
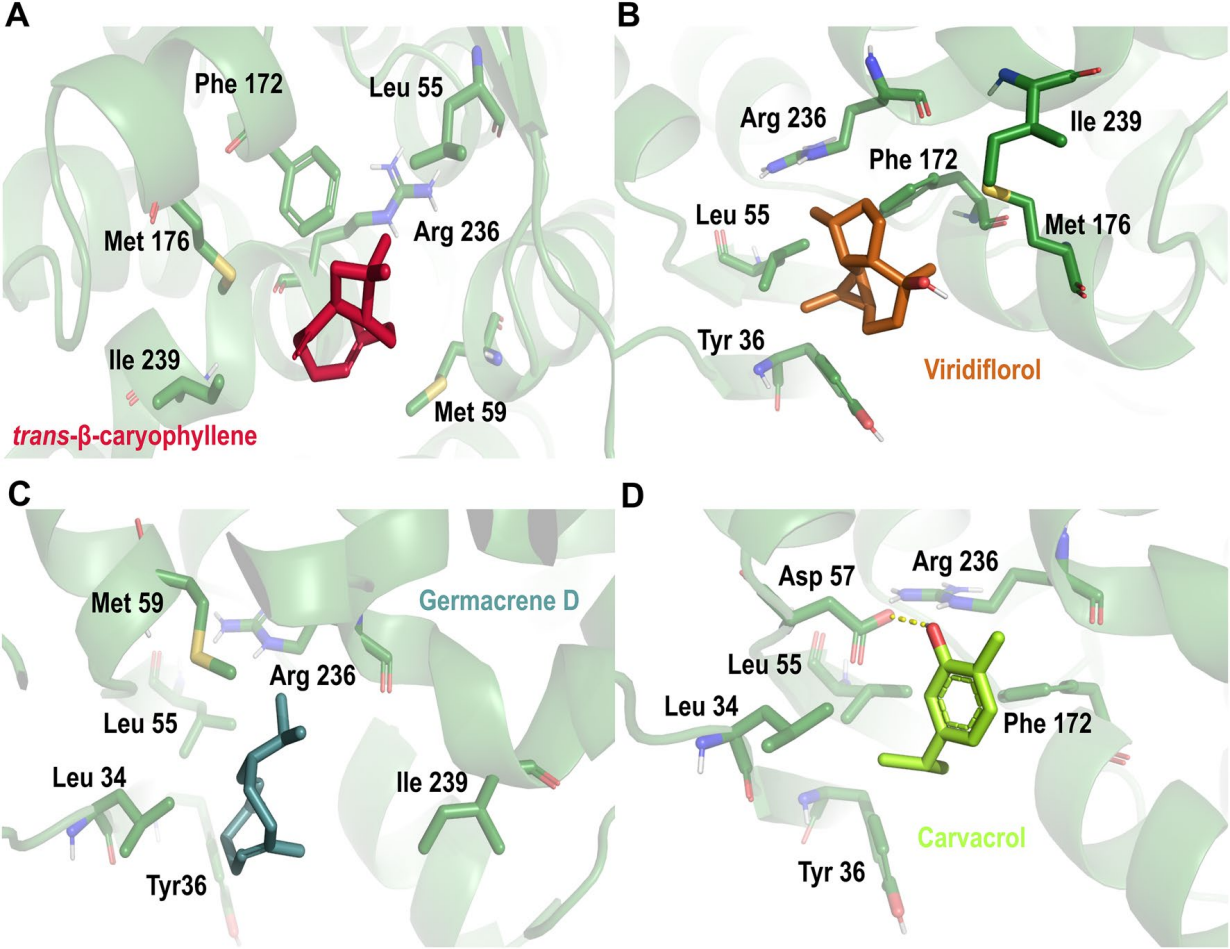
Docking poses of Best ranked secondary metabolites on subunit 5 of mitochondrial complex I in *Aedes aegypti*. (**A**) *trans-ꞵ*-caryophyllene; (**B**) viridiflorol; (**C**) Germacrene D; (**D**) carvacrol. Mitochondrial complex I’ residues are colored according to the atom type of the interacting amino-acid residues (protein’s carbon, forest green; oxygen, red; nitrogen, blue). The protein–ligand interactions are represented by dash lines as follows: hydrogen bond interactions are colored in yellow, π-π interactions are colored in blue.

Regarding mitochondrial complex I, *trans*-ꞵ-caryophyllene, vidiriflorol, germacrene D, and carvacrol were included in the top-ranked compounds. *trans*-ꞵ-Caryophyllene (docking score: − 5.8 kcal/mol, Table 2) is stabilized in a hydrophobic pocket made up of residues Ile 235-Ile 239, Leu 55-Met 59, Leu 34-Tyr 36, and Phe 172-Met 176, (Fig. 5A). Vidiriflorol (docking score − 5.5 kcal/mol, Table 2) is in a pocket formed by residues Met 59-Leu 55, Phe 172-Met 176, and Arg 236-Ile 239 (Fig. 5B). Docking results suggest a similar binding mode for germacrene D (docking score − 5.3 kcal/mol, Table 2) with a hydrophobic interaction with residues Ile 239-Ile235, Met 59-Leu 55, and Tyr 36-Leu 34 (Fig. 5C). Finally, carvacrol (docking score − 4.9 kcal/mol, Table 2) was also suggested by our docking results as a possible mitochondrial complex I inhibitor due to its binding pose within a pocket made up of residues Ile 235-Ile 239, Met 59-Leu 55, Leu 34-Tyr 36, π-π interaction with amino acid Phe 172, and H-bond interaction between residue Asp 57 and its hydroxyl group (Fig. 5D). Besides the above-mentioned secondary metabolites, *γ*-terpinene, *p*-cymene, thymol, *β*-pinene, limonene, and sabinene are also included within the top-ranked complex I as possible inhibitors according to the docking results obtained here (Table 2).

**Figure 6:**
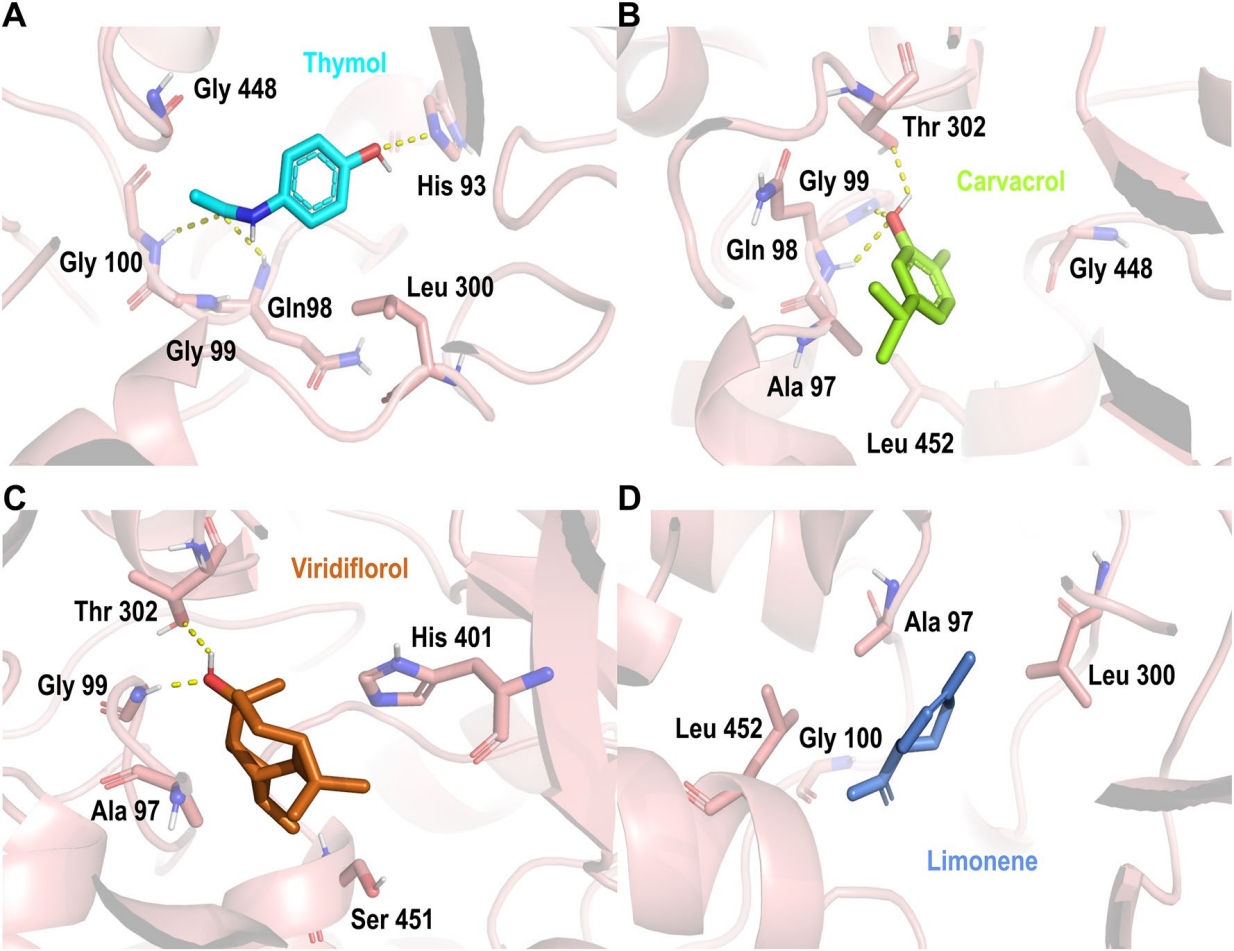
Docking poses of Best ranked secondary metabolites on subunit A of mitochondrial complex II in *A. aegypti*. (**A**) thymol; (**B**) carvacrol; (**C**) viridiflorol; (**D**) limonene. Mitochondrial complex II’ residues are colored according to the atom type of the interacting amino-acid residues (protein’s carbon, salmon; oxygen, red; nitrogen, blue). The protein–ligand interactions are represented by dash lines as follows: hydrogen bond interactions are colored in yellow.

To explore the binding mode and possible inhibitory effect of the in-house plant secondary metabolites database in mitochondrial complex II, we performed molecular docking in subunit A of mitochondrial complex II. As shown in Table 2, molecular docking suggests top-ranked secondary metabolites as possible inhibitors. First, thymol (docking score − 6.2 kcal/mol, Table 2) was stabilized in a binding pocket made up of residues Arg 446-Leu 452, His 93-Gly 100, making an H-bond interactions residues Gln 98, Gly 100, and His 93 (Fig. 6A). Carvacrol was also suggested as a possible mitochondrial complex II by our docking results, carvacrol (docking score − 5.8 kcal/mol, Table 2) is located within the same pocket formed by amino acid residues Arg 446-Leu 452, and His 93-Gly 100, generating an H-bond interaction between its hydroxyl group and Gly 100 and Thr 302 (Fig. 6B). Viridiflorol (docking score − 5.7 kcal/mol, Table 2) was located at the same binding pocket formed by amino acid residues His 93-Gly 100, and Arg 446-Leu 452, making an H-bond with residues Gly 99 and Thr 302 (Fig. 6C). Finally, in our top-ranked compounds against mitochondrial complex II, limonene (docking score − 5.4 kcal/mol, Table 2) was also included. Limonene’s binding model was proposed in the same pocket as previous secondary metabolites, with hydrophobic interactions with residues Leu 452-Arg 446, and Ala 96-Ile 101 (Fig. 6D). Besides above mentioned secondary metabolites, *γ*-terpinene, *p*-cymene, sabinene, *β*-pinene, *trans-β*-caryophyllene, and α-pinene were also proposed as possible mitochondrial complex II inhibitors (Table 2).

At the same time, our docking results suggest that flutolanil and succinate (a reported complex II inhibitors)^42^ were located in the same pocket. On the one hand, flutolanil is in a pocket formed of residues His 93-Gly 100, Leu 300-Glu 303, and Arg 446-Leu 455, having H-bond interactions with residues Gly 99, Gln 98, and Arg 446 (S. Fig. 21A). Meanwhile, succinate is in a pocket formed by amino acids Ala 96-Gly 100, Arg 446-Leu 452, having H-bond interactions with residues Arg 334, Gly 448, Gln 98, and Gly 99 (S. Fig. 21B).

**Figure 7:**
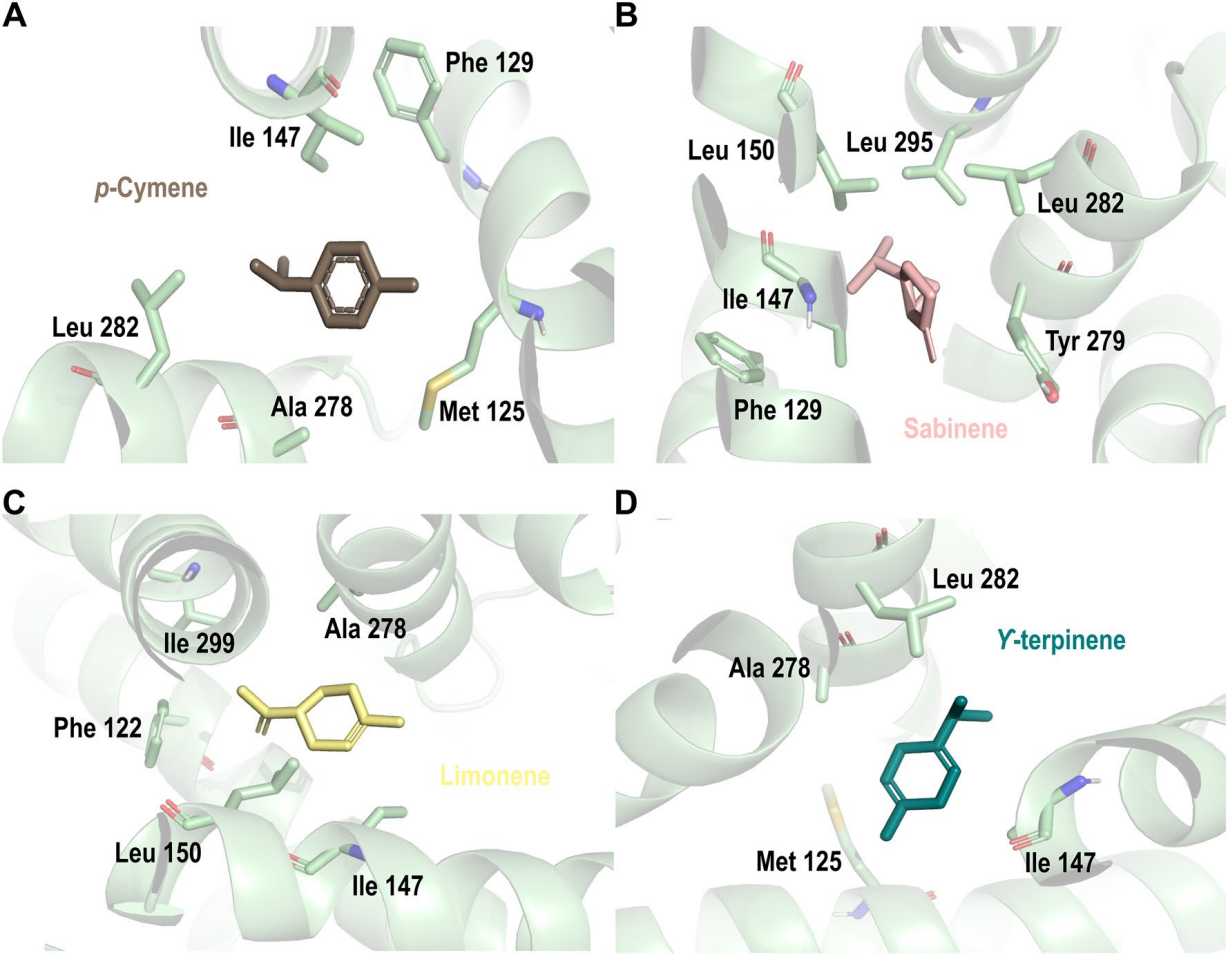
Docking poses of best ranked secondary metabolites on subunit A of mitochondrial complex III in *Aedes aegypti*. (**A**) *p*-cymene; (**B**) sabinene; (**C**) limonene; (**D**) γ-terpinene. Mitochondrial complex III’ residues are colored according to the atom type of the interacting amino-acid residues (protein’s carbon, pale green; oxygen, red; nitrogen, blue).

Same as the previous mitochondrial complexes, the mitochondrial complex III could be an essential target for insecticidal design due to its role in cytochrome reduction. For this, a virtual screening of plant secondary metabolites was performed to identify a new molecule with possible inhibitory activity in this mitochondrial complex. As shown in Table 2, *p-*cymene, sabinene, carvacrol, and γ-terpinene were top-ranked as possible mitochondrial complex III inhibitors. Our docking results in this work suggest that selected metabolites were located and stabilized by hydrophobic interactions in a pocket made up of residues Phe 129- Met 125, Phe 275-Leu 282, and Val 146-Leu 150 (Fig. 7).

Additionally, myxothiazol (docking score: − 6.03 kcal/mol), reported compound as a mitochondrial complex III inhibitor^43^ was located in the same pocket as the plant secondary metabolites studied here, a binding pocket made up of residues Phe 122-Phe 129, and Met 139-Leu 151 with an H-bond interaction between its amide group and Ile 147 (S. Fig. 22).

**Figure 8:**
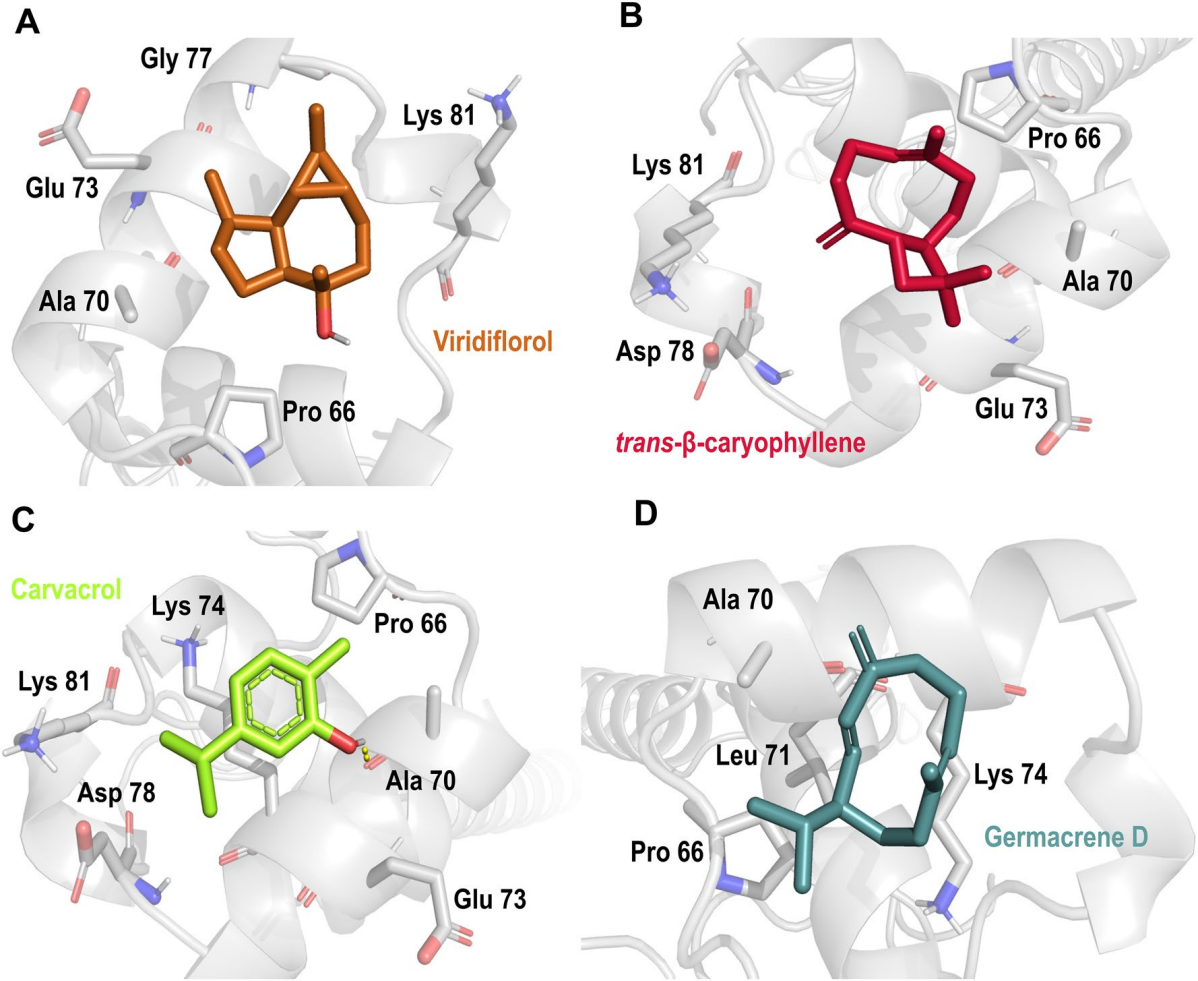
Docking pose of selected secondary metabolites on mitochondrial complex IV in *Aedes aegypti*. (**A**) viridiflorol; (**B**) *trans-*β-caryophyllene; (**C**) carvacrol; (**D**) germacrene D. Mitochondrial complex IV’ residues are colored according to the atom type of the interacting amino-acid residues (protein’s carbon, gray; oxygen, red; nitrogen, blue). The protein–ligand interactions are represented by dash lines as follows: hydrogen bond interactions are colored in yellow.

As a final receptor in the mitochondrial electron transport chain, complex IV was also evaluated aiming to identify possible insecticide compounds. As shown in Table 2, viridiflorol, carvacrol, *trans-β*-caryophyllene, and germacrene D may be involved in electron transport inhibition from cytochrome C to oxygen via complex IV. All the best-ranked compounds found here with possible inhibitory activity in complex IV were located in a binding pocket stabilized by hydrophobic interactions with amino acid residues Ala 70-Lys 81; besides, carvacrol was making H-bond interaction between its hydroxyl group and the amino acid residue Ala 70 (Fig. 8).

**Figure 9:**
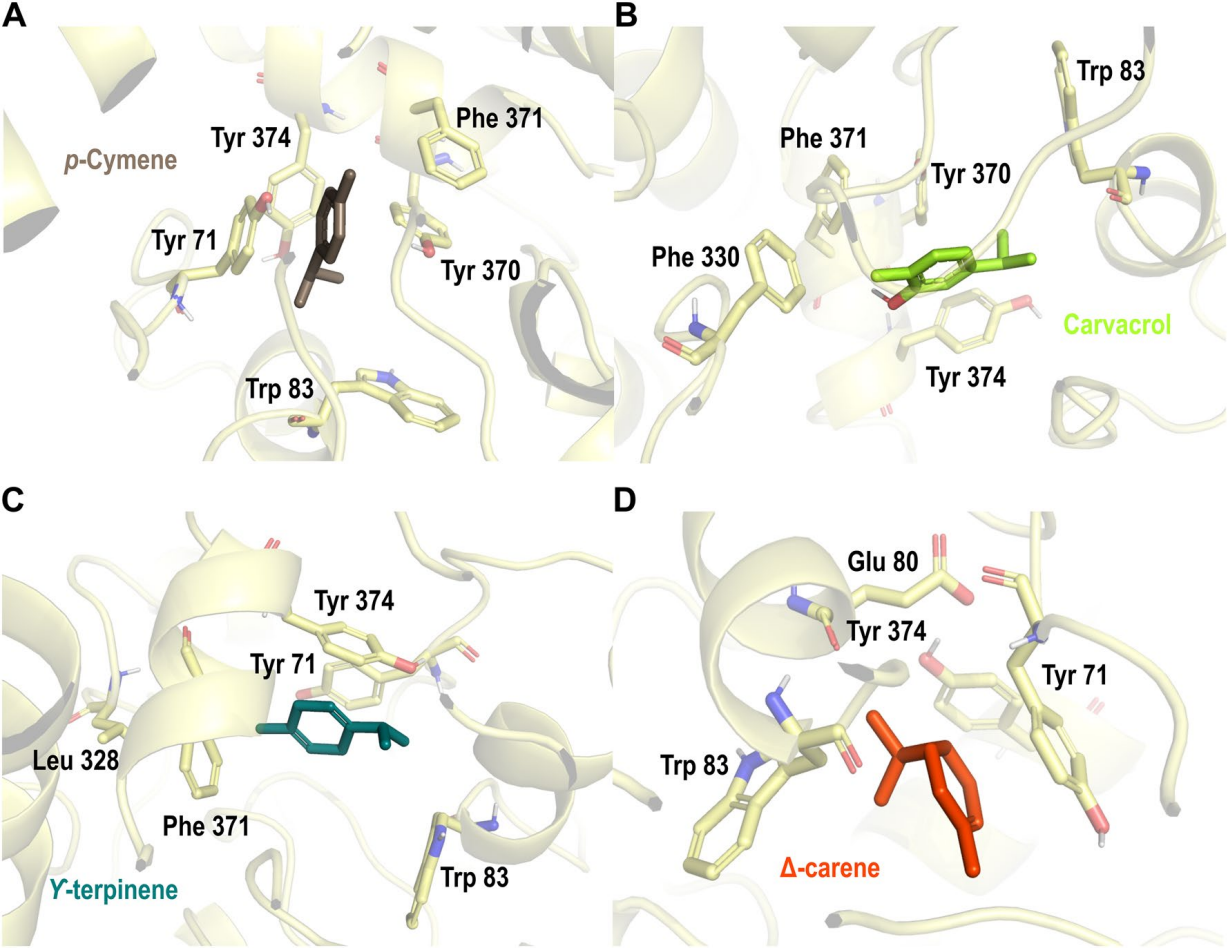
Docking poses of Best ranked plant secondary metabolites on the acetylcholinesterase crystal structure (PDBid: 1QON). (**A**) *p*-cymene; (**B**) carvacrol; (**C**) γ-terpinene; (**D**) Δ^3^-carene. Acetylcholinesterase' residues are colored according to the atom type of the interacting amino-acid residues (protein’s carbon, pale yellow; oxygen, red; nitrogen, blue). The protein–ligand interactions are represented by dash lines as follows: hydrogen bond interactions are colored in yellow, and -π interactions are colored in blue.

Besides mitochondria, acetylcholinesterase plays a key role in mosquito synapsis, making this enzyme an important target in insecticide design. Virtual screening was performed to explore the plant's secondary metabolites' potential inhibitory activity on acetylcholinesterase. As shown in Table 2, *p*-cymene, carvacrol, γ-terpinene, and Δ^3^-carene were the top-ranked compounds. All selected secondary metabolites were located in a binding pocket formed by amino acid residues Glu 80-Asn 84, Leu 328-Phe 330, Tyr 370-Asp 375, most of them stabilized by hydrophobic interactions. Meanwhile, carvacrol had additional π-π interactions with residues Tyr 374, Tyr 370, and Tyr 371 (Fig. 9).

Same as above mentioned plant secondary metabolites, reference compound I40 (docking score − 5.23 kcal/mol) was located within the same binding pocket and highly stabilized by π-π interactions with residues Tyr 374, Tyr 370, Tyr 371, and π-cation interaction with amino acid Trp 83 (S. Fig. 23).

## Discussion

Plant extracts and essential oils have been used and evaluated for years as insecticides against mosquitoes due to the ability of their compounds to affect multiple targets enhancing the insecticidal effect^44^. We studied the EOs distilled from American plants with larvicidal activity against *A. aegypti*, affecting electron transport through mitochondrial complexes and AChE. The possible binding mode of selected EO major compounds with molecular docking in targets such as mitochondrial subunits and acetylcholinesterase protein was proposed.

EOs have diverse biological activities against a broad spectrum of pests and microorganisms. They may act as insecticides, repellents, or anti-feed agents, affecting pests' growth and reproduction rate^45^. Additionally, the low EOs toxicity on mammals and their rapid environmental degradation make them an attractive alternative compared to conventional pesticides^46^. Here we report the insecticide activity against *A. aegypti* larvae of EOs from American native plants (Fig. 1). *S. aspera*, *T. diffusa*, *P. aduncum*, *L. origanoides (*chemotype thymol/carvacrol*)*, *L. origanoides* (chemotype carvacrol/thymol), *H. dilatata*, *E. quinquenervis*, and *C. moritzianus* had the best insecticide activity.

Since mitochondria are not only the powerhouse of the cell but also are involved in their activity deregulation in the cell death induction process and ROS production^47^. We evaluated the effect of EOs on electron transport through the mitochondrial complex aiming to know if insecticide activity was related to mitochondrial bioenergetic deregulation. NADH and succinate oxidase activity (Fig. 2) show the electron flux through the mitochondrial complexes generating a transmembrane potential that is directly coupled to ATP synthesis, raising a great interest in mitochondrial and the dysregulation of this organelle for insecticidal substances^3,18^. The activity of the mitochondrial ETC begins with the oxidation of substrates produced by processes such as the citric acid cycle, where NADH and succinate are primarily oxidized by complex I and II, respectively, and these electrons are transferred throughout the ETC until they reach oxygen, the final electron acceptor^48^. In this sense, the NADH and succinate oxidase activity inhibition by the EO components (Fig. 2) could be related to the blockade of electron transport along the mitochondrial complexes decreasing the ATP synthesis by inhibiting proton pumps^49,50^. Furthermore, this inhibition may also increase the reactive oxygen species (ROS) such as superoxide (O2^•−^), hydroxyl radical (HO^•^), and peroxide (H_2_O_2_), inducing damage in lipids, protein, and DNA, triggering cell death processes^49^.

Mitochondria and their electron transport complexes are an important target with wide applicability for the development of a new generation of insecticides, A virtual screening of principal metabolites include within the tested EO on key subunits in mitochondrial complexes I,II, III and IV as well as acetylcholinesterase was carried out^51,52^. We used computational approaches such as molecular docking to suggest a possible binding mode and inhibition of mitochondrial electron transport and its interactions with relevant residues. *C. moritzianus* EO had the highest inhibition of NADH oxidase activity, and its main metabolites include 1,8-cineole, limonene, *trans-β*-caryophyllene, and viridiflorol. The molecular docking results suggest important interactions of *trans-β*-caryophyllene and viridiflorol with complex I at ND5 (Fig. 5). Some authors reported that complex I is the largest redox-driven H^+^ pump in the mitochondrial chain, where amiloride derivatives 5-(N-methyl-N-isopropyl)amiloride (MIA), and 5-(N-ethyl-N-isopropyl)amiloride (EIPA) have been reported as complex I inhibitors due to their effect on quinone binding pocket^53,54^*.* Besides, Bourges et al. 2004 showed that inhibition of the absence of ND4 or ND5 subunits leads to a complete loss of NADH:ubiquinone oxidoreductase and NADH dehydrogenase activities^55^. Consequently, protein–ligand interactions predicted by molecular docking and the affinity of secondary metabolites to subunit5 may be related to the insecticide activity of the EOs tested (Fig. 5).

Molecular docking also suggests a secondary metabolites’ affinity with mitochondrial complexes II, III, IV, and acetylcholinesterase. The docking analysis agrees with Iaoka et al. 2015, who found that inhibitor flutolanil bounds near the quinone binding area with an H-bond interaction with three conserved residues in two different species (porcine and *A. suum*)^42^. Additionally, reported mitochondrial complex III inhibitor myxothiazol was found making H-bond interaction with residue Ile147 and hydrophobic interactions with relevant residues Phe275, Tyr274, and Phe 122 (S. Fig. 22), in the same pocket as the main EO components studied here (Fig. 7). We hypothesize those common interactions with key residues reported by Xiao et al. 2014 such as Phe 274, Tyr 275, and Tyr 131, and their homologs residues in *A. aegypti* with our secondary metabolites may be related to complex III inhibition and observed EO insecticide activity^56,57^.

It is important to verify the neurotoxic effect of new insecticide substances. AChE's function is to hydrolyze the neurotransmitter acetylcholine and thus regulate the neurotransmission^58^. Synthetic insecticides from the group of carbamates and organophosphates have AChE inhibition as their mechanism of action. These molecules have a single target of action, which has caused resistance in insect populations. On the other hand, essential oils with insecticidal activity may generate neurotoxic effects and affect multi targets. Previous studies showed that some compounds found in EOs had promising cholinesterase inhibitory activity, among them α-pinene, 1,8-cineole, and carvacrol^59^. Thymol and carvacrol inhibited the AChE of the susceptible and resistant strains of larvae of *Rhipicephalus (Boophilus) microplus* tick^60^. These phenolic monoterpenes were also components of *L. origanoides* EO evaluated in this work. All EOs studied here showed significant statistical differences from the negative control (Fig. 3).

Molecular docking analyses on acetylcholinesterase show common fingerprint interactions between reported inhibitor I40 (S. Fig. 23) and secondary metabolites (Fig. 9). Within these common interactions between secondary metabolites and inhibitor I40, it is possible to observe hydrophobic interactions with residues Tyr 71, Trp83, Tyr 370, Phe 371, Tyr 374, and His 380 located around the α-helix comprised of residues Thr 369- Asp 375, and the β-sheet formed by residues Ile 82-Trp 83 were observed and were in agreement with reported interactions in the literature^34,61^. Since the main function of the acetylcholinesterase is to remove the acetylcholine in the synaptic cleft, and the insecticidal activity caused by inhibition of this enzyme generates the death of the organism, the interactions with the residues Tyr 71, Trp 83, Tyr 370, and Tyr 374 could inactivate the insect AChE. The EOs with the higher inhibition of AChE activity *E. quinquenervis* and *H. dilatata* include Δ-carene within their principal components, and germacrene D and *trans*-β-caryophyllene as their major components. Karimi et al. 2021 reported a binding pose and inhibitory activity on AChE for Δ^3^-carene from *Cannabis sativa* with hydrophobic interactions with amino acid residues in the catalytic triad Trp 83, Phe 329, and Tyr 333, similar to interactions observed in this work (Fig. 9)^62,63^.

## Conclusion

Essential oils distilled from American plants exhibit similar or higher larvicidal activity than commercial insecticide temephos. Due to the comprehensive biological activities of EOs and a broad spectrum of pests and microorganisms, low toxicity and their rapid environmental degradation make them an attractive alternative to include within insecticide formulation. Overall, computational approaches such as homology models and molecular docking provide new insights into phytochemical compounds and their potential biological activities, generating great potential for developing and formulating novel and effective natural insecticides. Our results provide new insights into the possible mechanism of action of EOs and their major compounds for insecticide design, targeting not only the AChE activity as commercial insecticides and the *A. aegypti* mitochondria for effective and safe products, as observed in EO tested in this work. Additional biological assays are ongoing to determine each metabolite's biological effect against the targets proposed here.

## Supplementary Information

Supplementary Information.

## Data Availability

This article provides supplementary information. Additionally, the data that support the findings of this research are included in this paper. Further requirements can be directed to the corresponding author.
