# Supplementary material for: Insecticidal activity of essential oils from American native plants against Aedes aegypti (Diptera: Culicidae): an introduction to their possible mechanism of action
Source: Sci Rep. 2023 Feb 20;13:2989. doi: 10.1038/s41598-023-30046-8 (PMC9941582; doi:10.1038/s41598-023-30046-8)
Supplement: Supplementary file 1 — Supplementary Information. [file 41598_2023_30046_MOESM1_ESM.docx]

**Insecticidal activity of essential oils from American native plants against *Aedes aegypti* (Diptera: Culicidae): An introduction to their possible mechanism of action**

**Jonny E. Duque^1*^, Diana Urbina Duitama^2^, Luis C. Vesga^2^, Luis Ortíz Rodríguez^1^, Thomas S. Vanegas^1^, Elena E. Stashenko^3^  & Stelia C. Mendez- Sanchez^2^**

1. Centro de Investigaciones en Enfermedades Tropicales - Cintrop. Escuela de Medicina, Departamento de Ciencias Básicas. Universidad Industrial de Santander, Parque Tecnológico y de Investigaciones Guatiguará Km 2 El Refugio Piedecuesta, Santander, Colombia. A.A. (P.O. Box) 678 Bucaramanga, Colombia. Telephone: +57 (7) 634 4000 Ext 3503.
2. Grupo de Investigación en Bioquímica y Microbiología (GIBIM). Escuela de Química, Universidad Industrial de Santander, Bucaramanga A.A. 678, Colombia. Telephone: +57 (7) 634 4000 Ext 3584.
3. Centro de Investigación en Biomoléculas – CIBIMOL y Centro Nacional de Investigación para la Agroindustrialización de Plantas Aromáticas y Medicinales Tropicales – CENIVAM, Universidad Industrial de Santander, Bucaramanga, Colombia

***Corresponding author:** [jonedulu@uis.edu.co](mailto:jonedulu@uis.edu.co)

**S. Table 1.** Standard substances employed for essential oil characterization

| **N°** | **Compound** | **Formula** | **Classification** | **CAS** | **Purity, %** | **Source** |
| --- | --- | --- | --- | --- | --- | --- |
| 1 | Hexanal | C6H12O | DH | 66-25-1 | 98 |  |
| 2 | α-Pinene | C10H16 | HM | 80-56-8 | 98 |  |
| 3 | Camphene | C10H16 | MO | 79-92-5 | 95 |  |
| 4 | Sabinene | C10H16 | HM | 3387-41-5 | 95 |  |
| 5 | 1-Octen-3-ol | C8H16O | DH | 3391-86-4 | 98 |  |
| 6 | 6-Methyl-5-hepten- 2-one | C8H14O | DH | 110-93-0 | 98 |  |
| 7 | β-Myrcene | C10H16 | HM | 123-35-3 | 94 |  |
| 8 | α-Phellandrene | C10H16 | HM | 99-83-2 | 98 |  |
| 9 | α-Terpinene | C10H16 | HM | 99-86-5 | 97 |  |
| 10 | *p-*Cymene | C10H14O | MO | 99-87-6 | 99 |  |
| 11 | Limonene | C10H16 | HM | 138-86-3 | 97 |  |
| 12 | 1,8-Cineole (eucaliptol) | C10H18O | SO | 470-82-6 | 99 |  |
| 13 | γ-Terpinene | C10H16 | HM | 99-85-4 | 96 |  |
| 14 | Linalool | C10H18O | MO | 78-70-6 | 97 |  |
| 15 | (*Z)*-Thujone | C10H16O | MO | 546-80-5 | 98 |  |
| 16 | *(Z)*-Limonene oxide | C10H16O | MO | 13837-75-7 | 97 | *Sigma*  *Aldrich* (*St. Louis*, MO, EE. UU.) |
| 17 | Citronelal | C10H18O | MO | 106-23-0 | 90 |  |
| 18 | Isopulegol | C10H18O | MO | 89-79-2 | 99 |  |
| 19 | Menthone | C10H18O | MO | 89-80-5 | 98,5 |  |
| 20 | Isomenthone | C10H18O | MO | 14073-97-3 | 90 |  |
| 21 | Menthol | C10H20O | MO | 2216-51-5 | 99 |  |
| 22 | Terpinen-4-ol | C10H18O | MO | 562-74-3 | 95 |  |
| 23 | 4-Allylanisole | C10H12O | MO | 140-67-0 | 98 |  |
| 24 | α-Terpineol | C10H18O | MO | 98-55-5 | 90 |  |
| 25 | (*Z*)-Dihydrocarvone | C10H16O | MO | 7764-50-3 | 98 |  |
| 26 | (*E*)-Dihydrocarvone | C10H16O | MO | 5524-05-0 | 98 |  |
| 27 | Citronelol | C10H20O | MO | 106-22-9 | 95 |  |
| 28 | Nerol | C10H18O | MO | 106-25-2 | 97 |  |
| 29 | (*Z*)-Carveol | C10H16O | MO | 1197-06-4 | 97 |  |
| 30 | Geraniol | C10H18O | MO | 106-24-1 | 98 |  |
| 31 | Carvone | C10H14O | MO | 99-49-0 | 96 |  |
| 32 | Geranial | C10H16O | MO | 141-27-5 | 95 |  |
| 33 | (*E*)-  Cinnamaldehyde | C9H8O | HO | 14371-10-9 | 98 |  |

DH: Hydrocarbon derivative, HO: Oxygenated hydrocarbon, HM Monoterpene hydrocarbon, MO Oxygenated monoterpene, HS Sesquiterpene hydrocarbon, SO Oxygenated sesquiterpene.

**S. Table 2.** Chemical characterization of essential oils from collected plants studied in this work

| **EO code** | **Scientific name** | **Major components (Relative GC area, %)** |
| --- | --- | --- |
| 001 | *Steiractinia aspera* Cuatrec.  (Asteraceae) | α-pinene (24.9%), β-pinene (14.8%), germacrene D (13.1%), β-phellandrene (10.1%), α-phellandrene (6.3%), sabinene (4.6%), *p*-cymene (4.5%), *trans*-β-caryophyllene (3.1%); α-copaene (2.6%), limonene (2.4%). |
| 002 | *Turnera diffusa* Willd.  (Passifloraceae) | Dehidrofukinone (25.4%), aristolochene (17.9%), valencene (7.4%), β-selinene (5.2%), *trans*-β-caryophyllene (4.0%), β-elemene (4.0%), premnaspirodiene (3.7%), guaiol (3.5%), germacra-4,5,10-trien-1-α-ol (3.5%), caryophyllene oxide (3.3%). |
| 003 | *Lippia origanoides* Kunth, Chemotype phellandrene  (Verbenaceae) | *trans*-β-caryophyllene (18.6%), α-humulene (10.1%), α-phellandrene (9.3%), *p*-cymene (8.7%), 1,8-cineole (6.5%), limonene (4.4%), caryophyllene oxide (3.8%), β-phellandrene (3.1%), camphene (2.5%), germacrene D (2.2%). |
| 004 | *Calycolpus moritzianus* (O.Berg) Burret.  (Myrtaceae) | 1,8-cineole (19.1%), limonene (17.6%), *trans*-β-caryophyllene (6.3%), viridiflorol (5.7%), α-pinene (5.1%), *trans*,*trans*-geranyl linalool (4.0%), *trans*-nerolidol (3.5%), α-copaene (3.2%), selina-3,7(11)-diene (2.8%), viridiflorol (2.7%). |
| 005 | *Piper aduncum* L.  (Piperaceae) | Piperitone (14.8%), *trans*-β-Caryophyllene (7.4%), viridiflorol (6.5%), limonene (6.0%), δ-cadinene (5.5%), α-pinene (4.6%), α-phellandrene (4.4%), caryophyllene oxide (3.8%), 1,8-cineole (3.6%), *p*-cymene (3.0%). |
| 006 | *Elaphandra quinquenervis* (S.F.Blake) H.Rob.  (Asteraceae) | Germacrene D (20.7%), α-phellandrene (9.1%), α-pinene (6.8%), *trans*-β-caryophyllene (5.1%), Δ3-carene (4.9%), limonene (4.5%), β-cubebene (3.5%), α-humulene (2.6%), premnaspirodiene (2.6%), δ-cadinene (2.6%). |
| 007 | *Hyptis dilatata* Benth.  (Lamiaceae) | *trans*-β-Caryophyllene (20.2%), alcanfor (16.1%), 3-Δcarene (15.5%), α-pinene (10.5%), palustrol (8.7%), α-gurjunene (4.7%), ledol (3.4%), limonene (2.4%), camphene (1.7%), viridiflorol (1.5%), aromadendrene (1.5%). |
| 008 | *Lippia origanoides* Kunth. Chemotype carvacrol+*p*-cymene  (Verbenaceae) | Carvacrol (35%), *p*-cymene (14.4%), thymol (8.0%), γ-terpinene (5.3%), *trans*-β-caryophyllene (4.4%), β-myrcene (2.4%), carvacryl acetate (2.0%), thymol methyl ether (1.9%), α-terpinene (1.7%). |
| 009 | *Lippia origanoides* Kunth. Chemotype *trans*-β-caryophyllene  (Verbenaceae) | *trans*-β-caryophyllene (15.1%), thymol (14%), 1,8-cineole (13%), *p*-cymene (12.6%), α-humulen (8.1%), α-phellandrene (7.1%), α-eudesmol (2.6%), caryophyllene oxide (2.5%), γ-terpinene (2.4%), limonene (2.1%). |
| 010 | *Lippia origanoides* Kunth. Chemotype thymol  (Verbenaceae) | Thymol (75.3%), *trans*-β-Caryophyllene (5.4%), carvacrol (4.9%), α-humulen (3.2%), p-cymene (2.3%), thymol acetate (1.6%), thymol methyl ether (1.3%), caryophyllene oxide (1.3%), *trans*-β-bergamotene (1.0%). |
| 011 | *Turnera diffusa* Willd.  (Passifloraceae) | Aristolecheno (20.9%), dehidrofukinona (19.3%), valencene (6.5%), β-selinene (5.8%), β-elemene (5.0%), *trans*-β-caryophyllene (4.9%), premnaspirodiene (4.7%), p-cymene (3.6%), germacra-4,5,10-trien-1-α-ol (3.6%), guaiol (3.3%). |
| 012 | *Satureja viminea* L.  (Labiatae) | *p*-Ment-3-en-8-ol (32.4%), pulegone (16.1%), *trans*-9-epi-caryophyllene (8.9%), *trans*-β-caryophyllene (8.4%), caryophyllene oxide (4.3%), spathulenol (3.6%), benzyl benzoate (2.4%), δ-Cadinene (2.2%), *trans*-pulegol (1.8%), p-Mentha-3,8-diene (1.5%). |
| 013 | *Psidium sartorianum Nied.*  (Myrtaceae) | *trans*-β-Caryophyllene (12.7%), caryophyllene oxide (12.0%), N.I. C15H24O (8.8%), dehydrofukinone (7.5%), caryophylla-4(12),8(13)-dien-5-β-ol (4.8%), germacrene B (4.1%), 1,8-cineole (3.7%), *p*-cymene (2.9%), β-pineno (2.7%), Selina-3,7(11)-diene (2.5%), β-selinene (2.1%), premnaspirodiene (2.0%). |
| 014 | *Varronia curassavica* Vell.  (Boraginaceae) | *trans*-β-Caryophyllene (19.2%), germacrene D (12.3%), *trans*-β-Guaiene (11.8%), α-pinene (9.4%), α-copaene (7.0%), β-pineno (4.1%), biciclogermacreno (3.9%), β-elemene (2.8%), δ-cadinene (2.8%), α-humulen (2.7%). |
| 015 | *Ocimum basilicum* L.  (Labiatae) | Linalool (42.7%), estragole (18.6%), 1,8-cineole (8.1%), Germacrene D (4.9%), e-α-cadinol (4.2%), γ-cadinene (3.7%), α-humulen (2.5%), β-elemene (2.2%), biciclogermacreno (2.2%), *trans*-α-bergamotene (1.1%). |
| 016 | *Calycolpus moritzianus* (O.Berg) Burret.  (Myrtaceae) | 1,8-Cineole (15.4%), limonene (14.7%), viridiflorol (7.1%), *trans*-*trans*-geranyl linalool (6.7%), *trans*-β-caryophyllene (6.2%), β-selinene (5.8%), *trans*-Nerolidol (4.0%), α-pinene (3.5%), selina-3,7(11)-diene (3.0%), α-copaene (3.0%). |
| 017 | *Turnera diffusa* Willd.  (Passifloraceae) | Aristolochene (20.6%), dehidrofukinona (17.3%), p-cymene (5.8%), β-selinene (5.6%), valencene (5.2%), premnaspirodiene (4.2%), caryophyllene oxide (3.6%), *trans*-β-caryophyllene (2.8%), germacra-4,5,10-trien-1α-ol (2.4%), α-selinene. |
| 018 | *L. origanoides* Kunth. chemotype Thymol + p-cymene  (Verbenaceae) | Thymol (49.4%), *p*-cymene (19.1%), γ-terpinene (9.2%), β-myrcene (5.2%), α-terpinene (2.9%), carvacrol (2.7%), thymol methyl ether (1.8%), *trans*-β-caryophyllene (1.6%), *cis*-β-ocimene (1.2%), limonene (0.9%). |
| 019 | *Lippia origanoides* Kunth. Chemotype Thymol  (Verbenaceae) | Thymol (71.7%), *p*-cymene (10.5%), carvacrol (4.4%), β-myrcene (2.1%), γ-terpinene (2.0%), caryophyllene oxide (1.6%), thymol methyl ether (0.9%), *trans*-β-caryophyllene (0.9%), humulene epoxide II (0.7%), terpinen-4-ol (0.7%). |
| 020 | *Lippia micromera* Schauer.  (Verbenaceae) | *p*-Cymene (26.8%), thymol methyl ether (26.3%), thymol (17.8%), thymol acetate (5.7%), γ-terpinene (5.4%), 1,8-cineole (5.1%),α-terpinene (2.0%), β-myrcene (2.0%), *trans*-β-caryophyllene (1.7%), α-thujene (1.3%), caryophyllene oxide (0.9%). |

**S. Table 3.** ADME properties of principal metabolites included within the essential oils

| Compound | ADME descriptors | | | | | | |
| --- | --- | --- | --- | --- | --- | --- | --- |
|  | MW | donadorHB | accptHB | LogPo/w | Violation | GI Absorption | BBB permeant |
| 1,8-cineol | 154.250 | 0.000 | 1.000 | 2.670 | 0 | High | Yes |
| α- Pinene | 136.230 | 0.000 | 0.000 | 3.440 | 1 | Low | Yes |
| α- Terpineol | 154.252 | 1.000 | 0.750 | 2.963 | 0 | High | Yes |
| α- Eudesmol | 222.370 | 1.000 | 0.750 | 4.029 | 0 | High | Yes |
| β- Pinene | 136.230 | 0.000 | 0.000 | 3.420 | 1 | Low | Yes |
| β- Elemene | 218.338 | 0.000 | 2.000 | 3.749 | 0 | Low | No |
| Carvacrol | 150.220 | 1.000 | 0.750 | 3.298 | 0 | High | Yes |
| Camphor | 152.236 | 0.000 | 2.000 | 1.943 | 0 | High | Yes |
| Carvotan acetone | 152.236 | 0.000 | 2.000 | 2.228 | 0 | High | Yes |
| Caryophylene oxide | 220.354 | 0.000 | 2.000 | 2.560 | 0 | High | Yes |
| Δ3-carene | 136.230 | 0.000 | 0.000 | 3.420 | 1 | Low | Yes |
| Estragole | 148.204 | 0.000 | 0.750 | 2.577 | 0 | High | Yes |
| γ-terpinene | 136.230 | 0.000 | 0.000 | 3.350 | 0 | Low | Yes |
| Geranyl acetate | 196.289 | 0.000 | 2.000 | 3.505 | 0 | High | Yes |
| Germacrene D | 204.350 | 0.000 | 0.000 | 4.300 | 1 | Low | No |
| Intermediol | 222.370 | 1.000 | 0.750 | 3.949 | 0 | High | Yes |
| Ledol | 222.370 | 1.000 | 0.750 | 3.985 | 0 | High | Yes |
| Limonene | 136.230 | 0.000 | 0.000 | 3.370 | 0 | Low | Yes |
| Linalool | 154.252 | 1.000 | 0.750 | 3.173 | 0 | High | Yes |
| *p*-Menth-3-en-8-ol | 168.278 | 1.000 | 1.700 | 2.705 | 0 | High | Yes |
| *p*-Cymene | 134.221 | 0.000 | 0.000 | 3.668 | 0 | Low | Yes |
| Palustrol | 222.370 | 1.000 | 0.750 | 4.011 | 0 | High | Yes |
| Piperitone | 152.236 | 0.000 | 2.000 | 2.184 | 0 | High | Yes |
| Pulegone | 152.236 | 0.000 | 2.000 | 2.226 | 0 | High | Yes |
| Sabinene | 136.230 | 0.000 | 0.000 | 3.250 | 1 | Low | Yes |
| Spathulenol | 220.354 | 1.000 | 0.750 | 3.921 | 0 | High | Yes |
| Viridiflorol | 222.370 | 1.000 | 0.750 | 3.952 | 0 | High | Yes |
| *trans*-β-caryophyllene | 204.350 | 0.000 | 0.000 | 4.240 | 1 | Low | No |
| Thymol | 150.220 | 1.000 | 0.750 | 3.299 | 0 | High | Yes |
| Thymol acetate | 152.236 | 0.000 | 2.000 | 2.184 | 0 | High | Yes |
| Thymol methyl ether | 164.247 | 0.000 | 0.750 | 3.287 | 0 | High | Yes |

**S. Figure 1.** GC/MS of *Steiractinia aspera* EO code 001


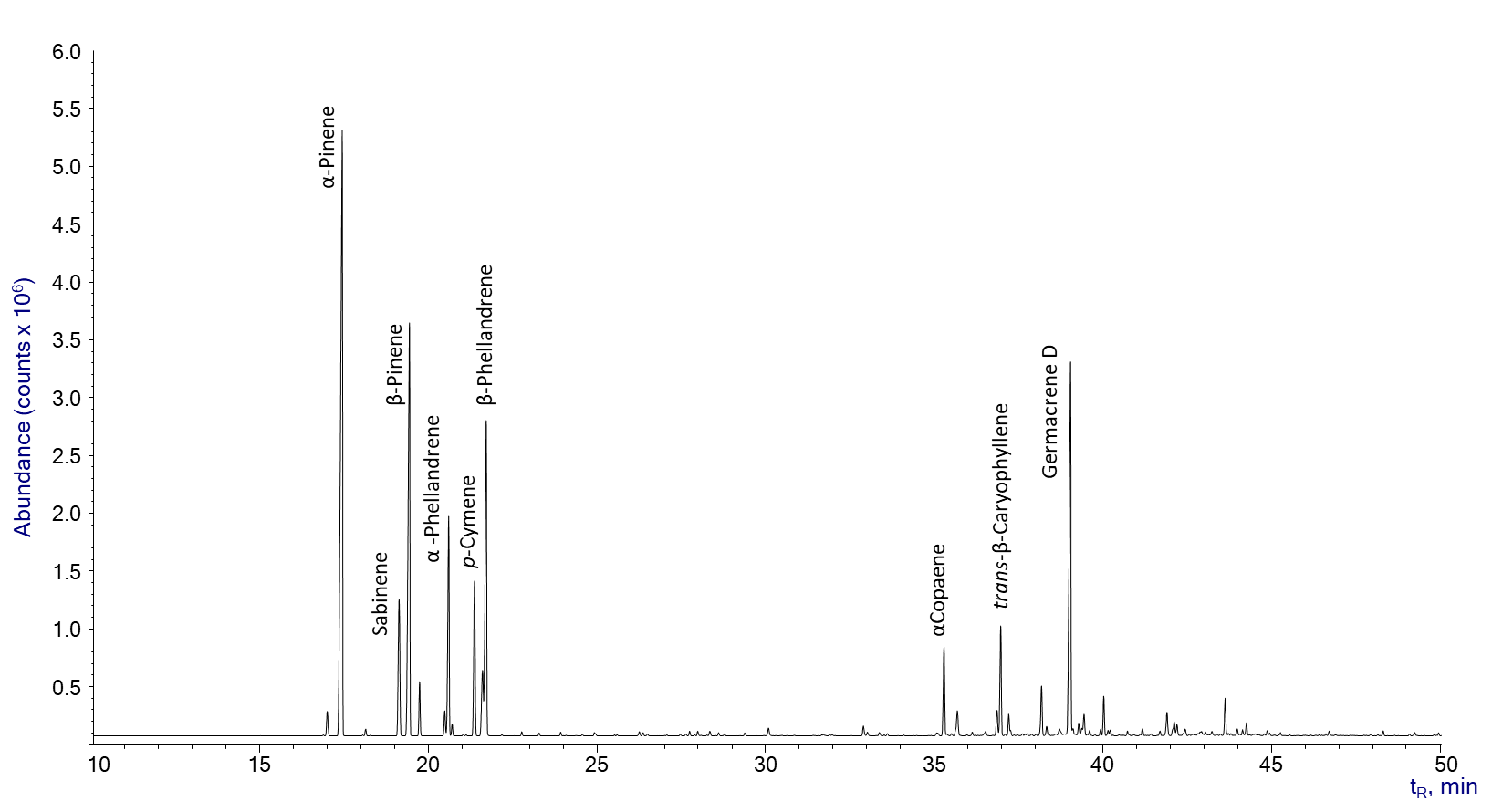


**S. Figure 2.** GC/MS of *Turnera diffusa* EO code 002


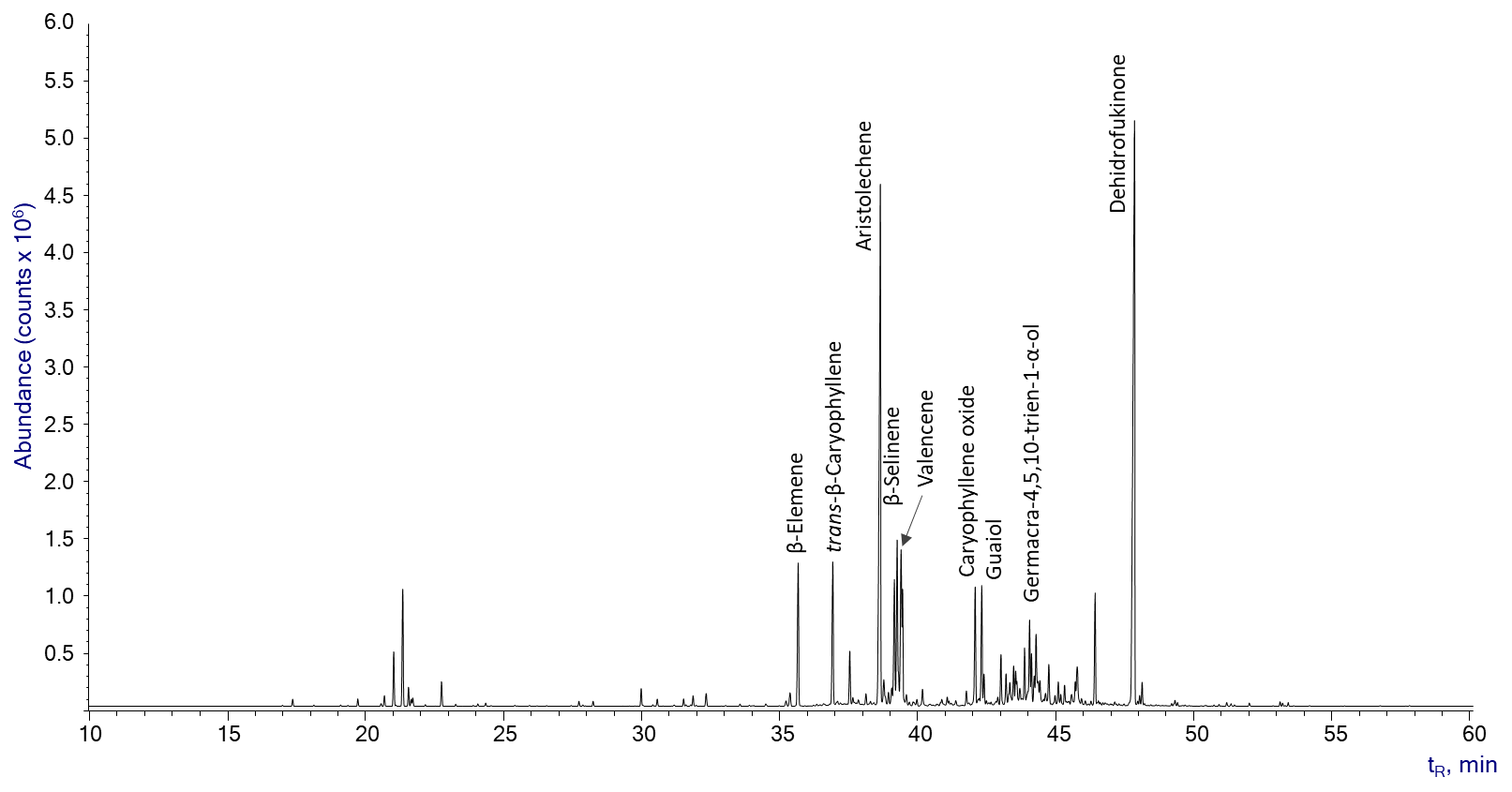


**S. Figure 3.** GC/MS of *Lippia origanoides,* chemotype felandrene EO code 003


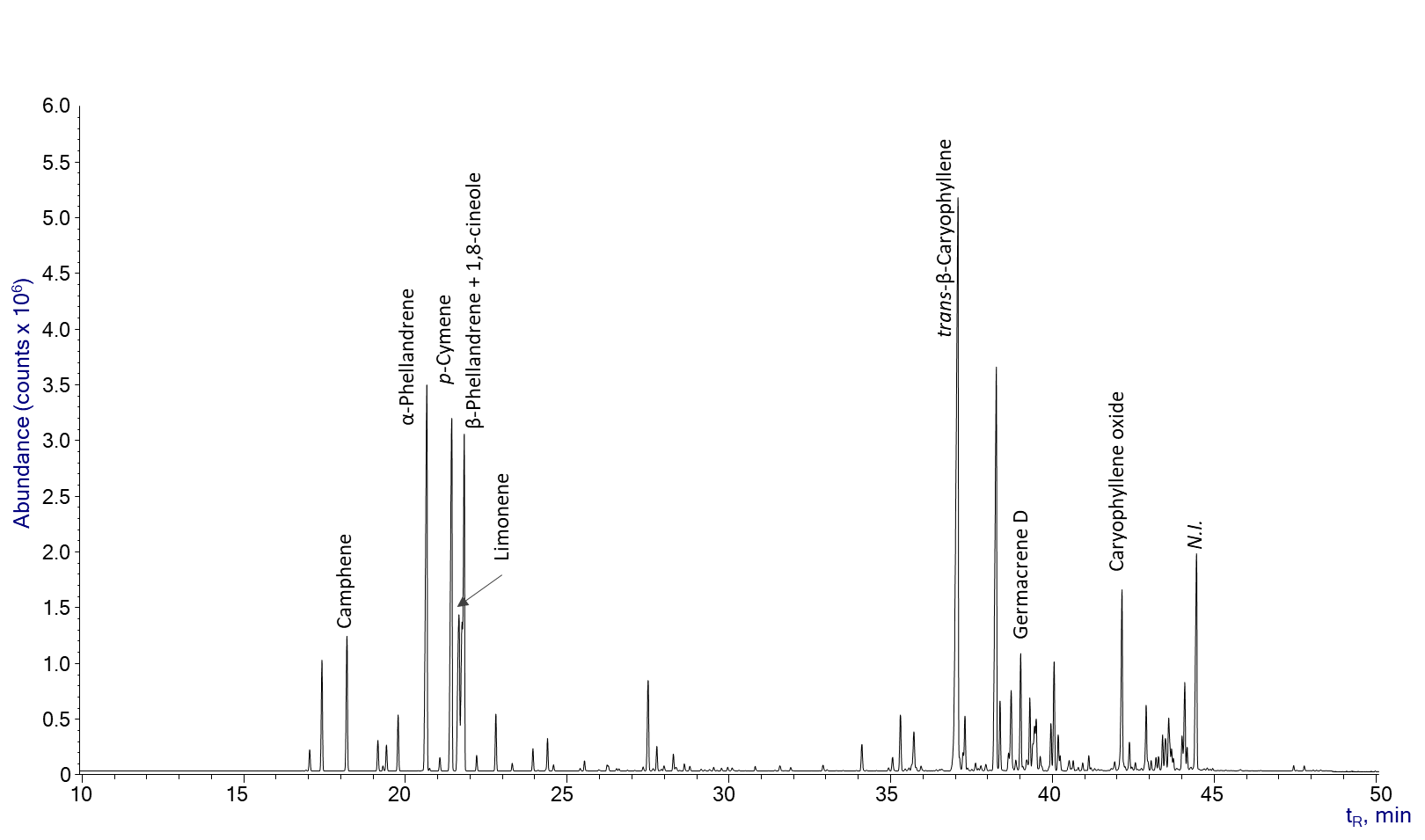


**S. Figure 4.** GC/MS of *Calycolpus maritzianus* EO code 004


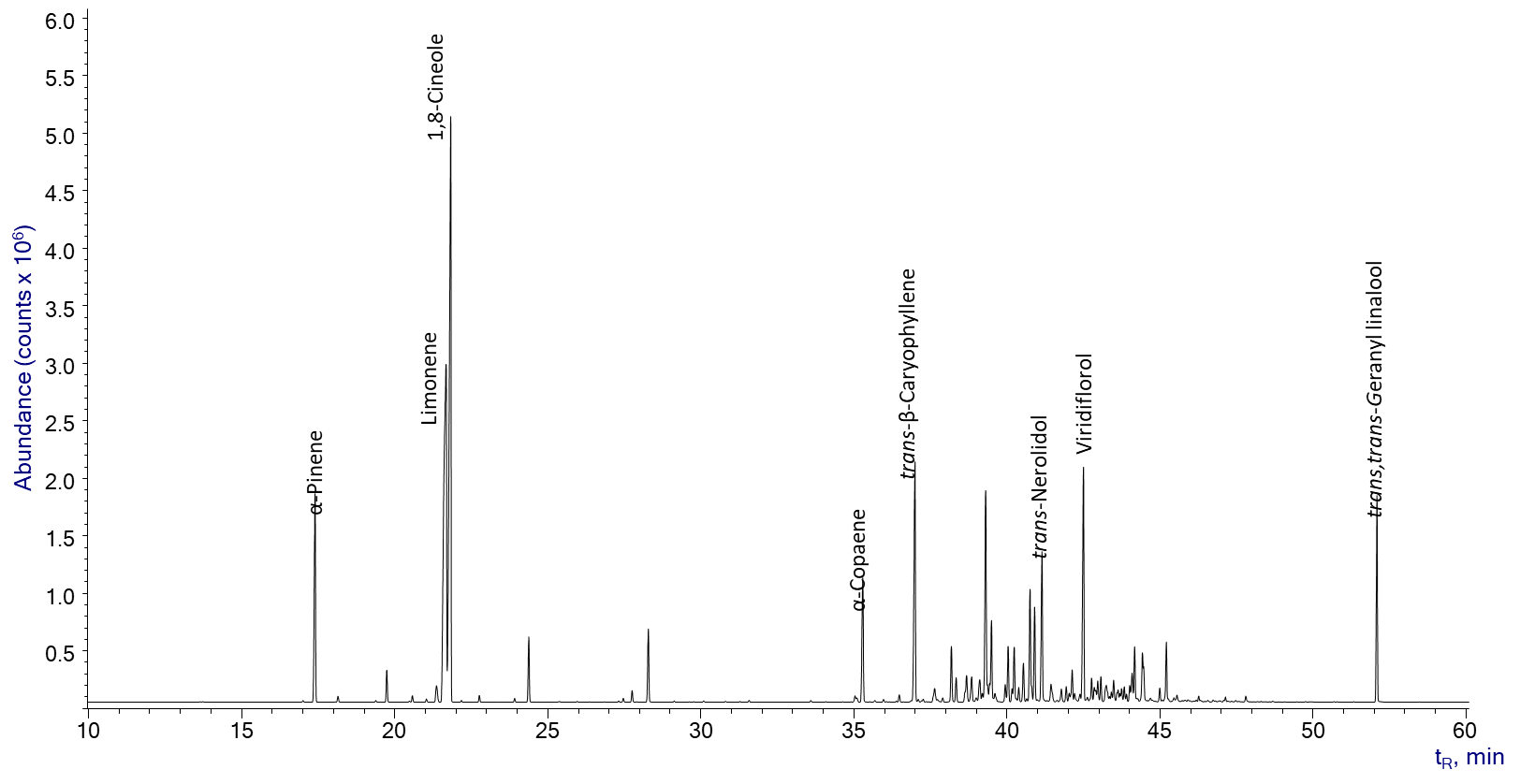


**S. Figure 5.** GC/MS of *Piper aduncum* EO code 005


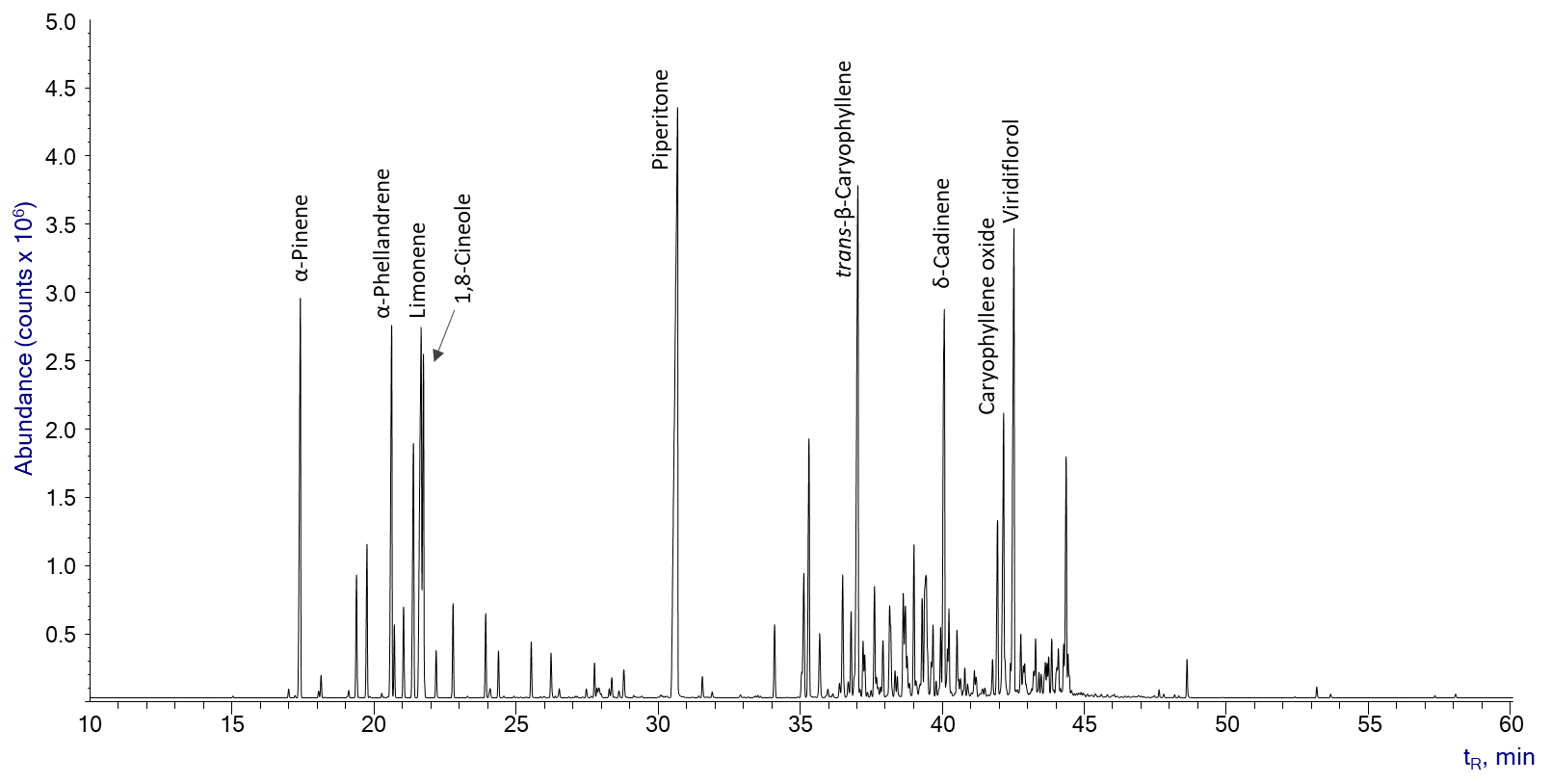


**S. Figure 6.** GC/MS of *Elaphandra quinquenervis* EO code 006


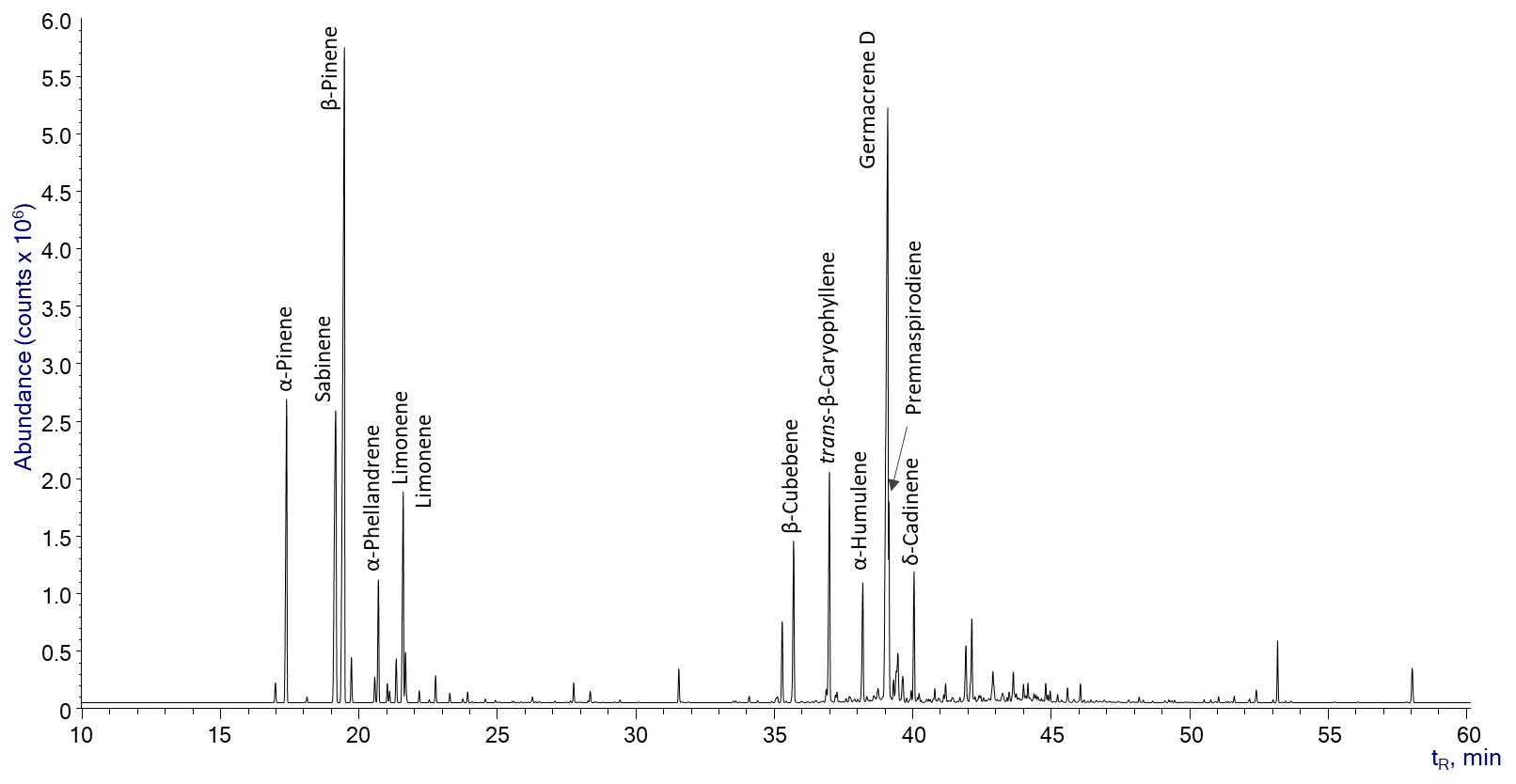


**S. Figure 7.** GC/MS of *Hyptis dilatata* EO code 007


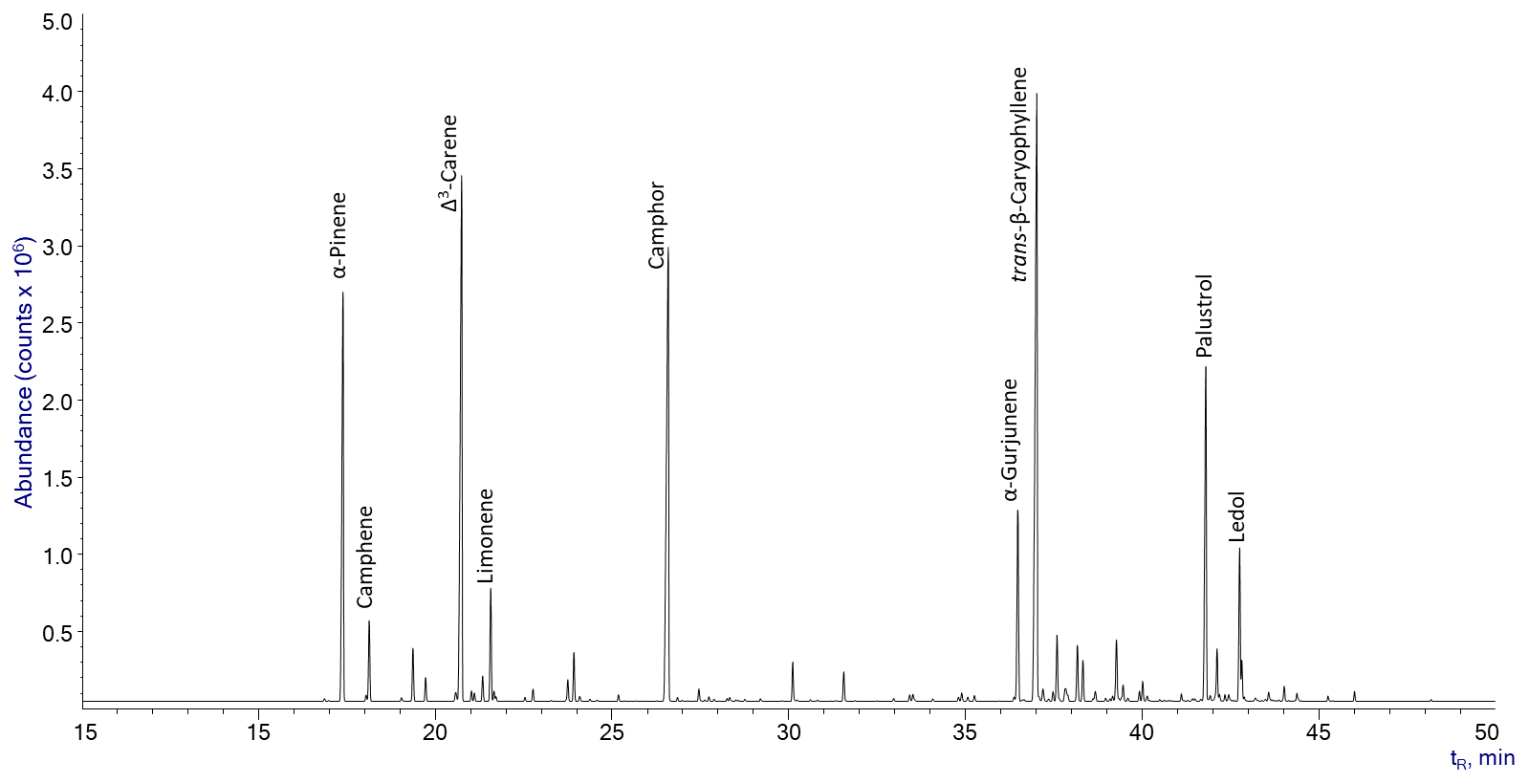


**S. Figure 8.** GC/MS of *Lippia origanoides chemotype carvacrol* EO code 008


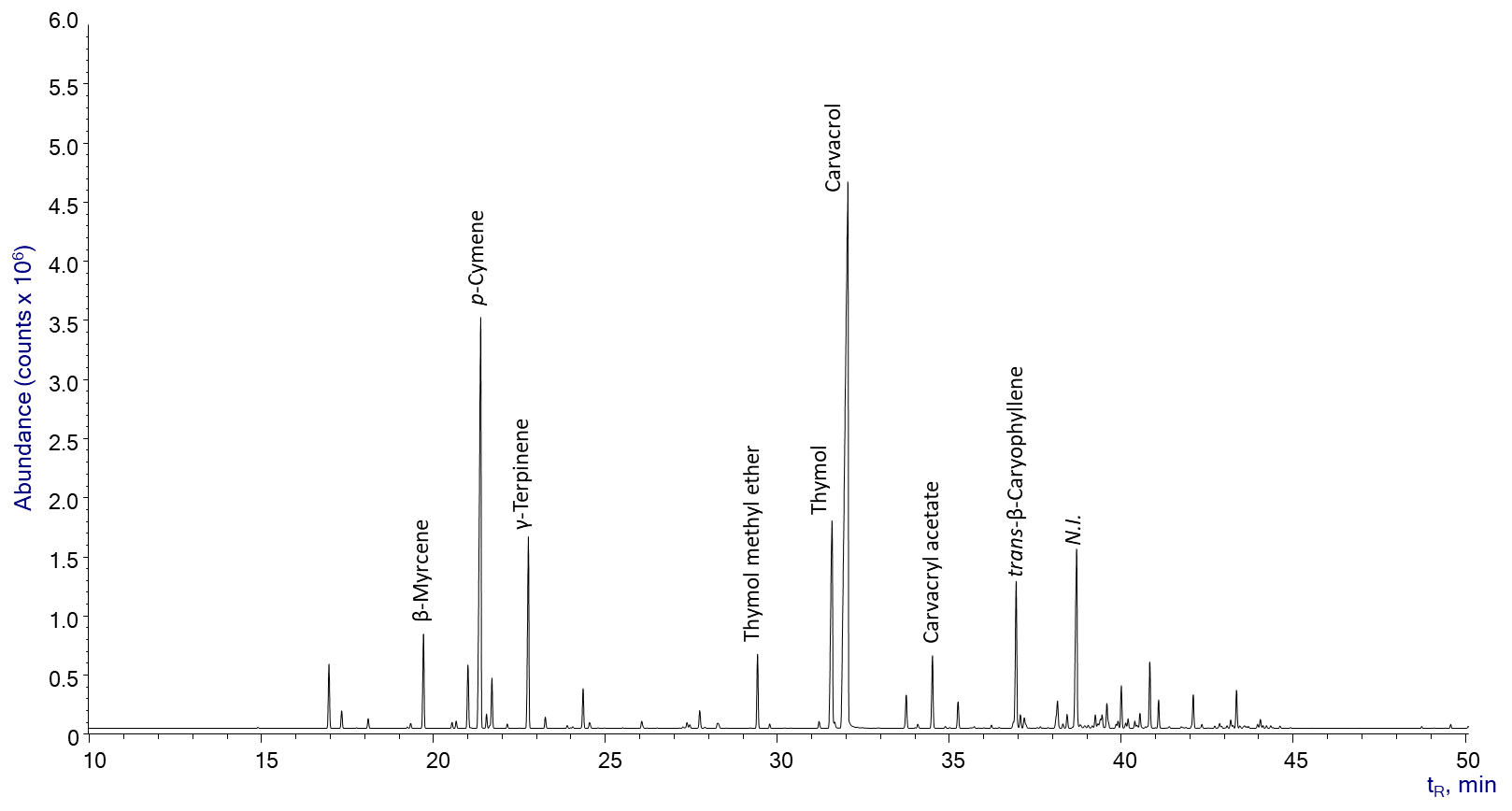


**S. Figure 9.** GC/MS of *Lippia origanoides chemotype trans-β-cariofilene* EO code 009


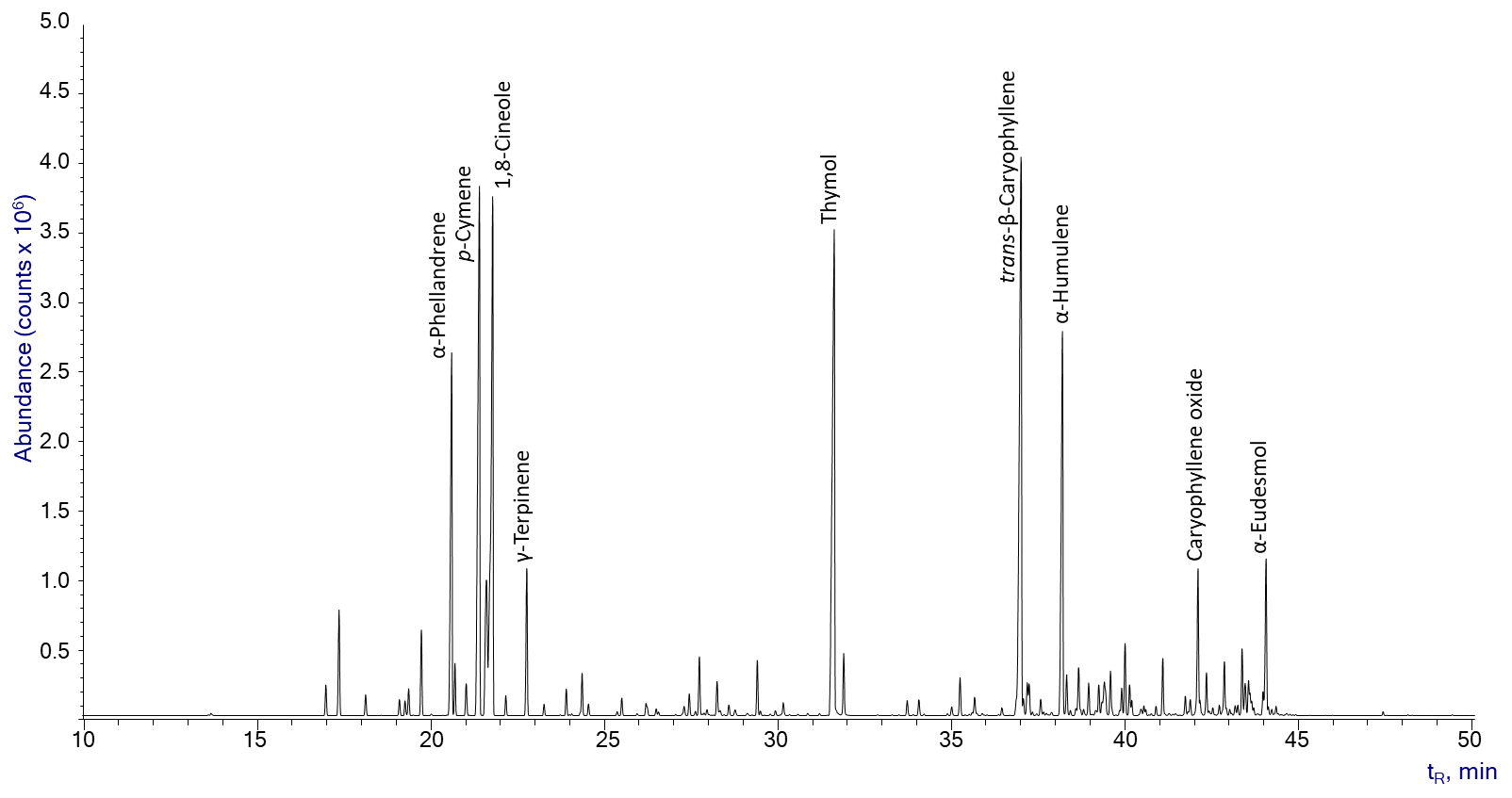


**S. Figure 10.** GC/MS of *Lippia origanoides chemotype thymol* EO code 0010


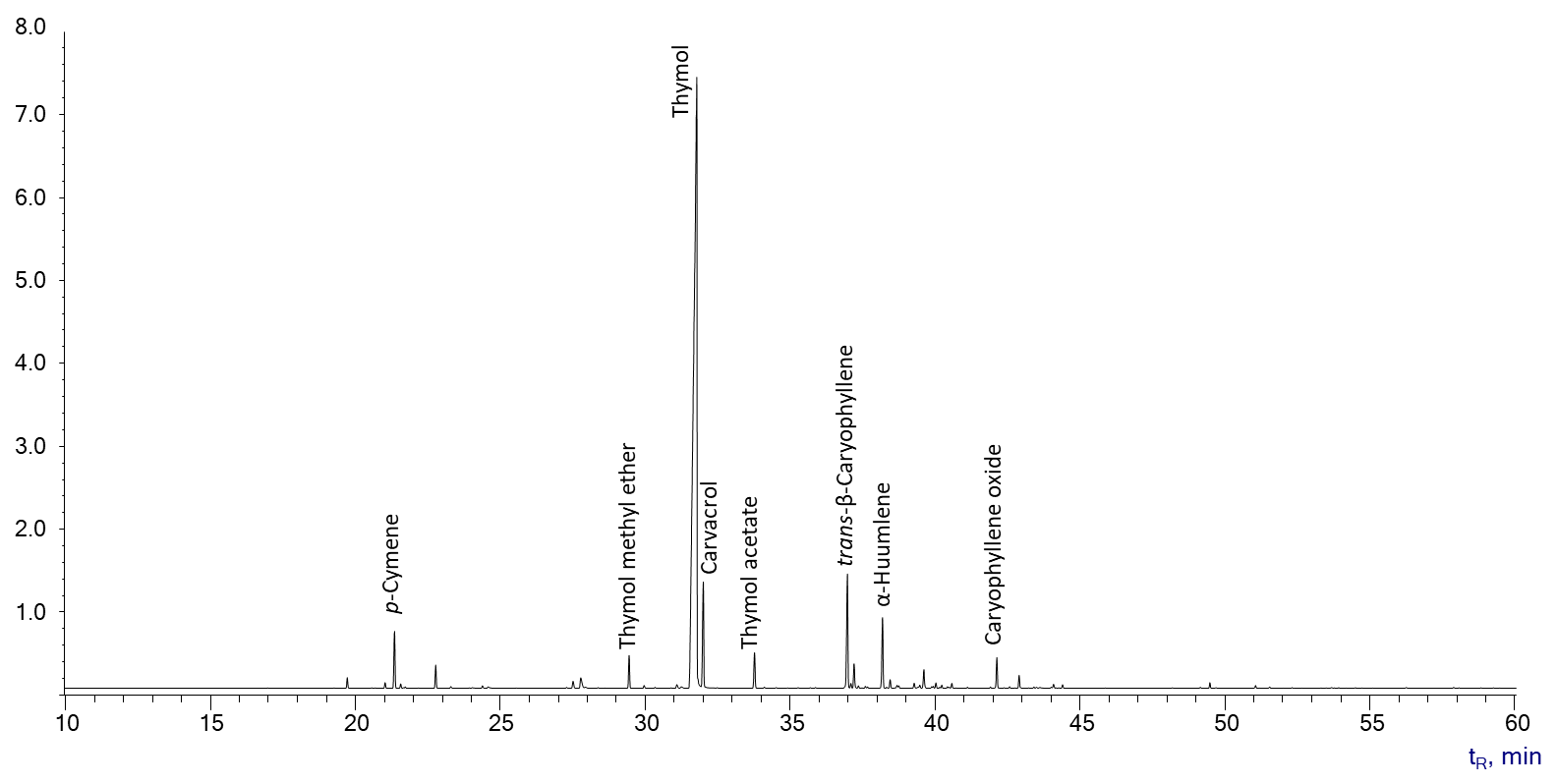


**S. Figure 11.** GC/MS of *Turnera diffusa* EO code 0011


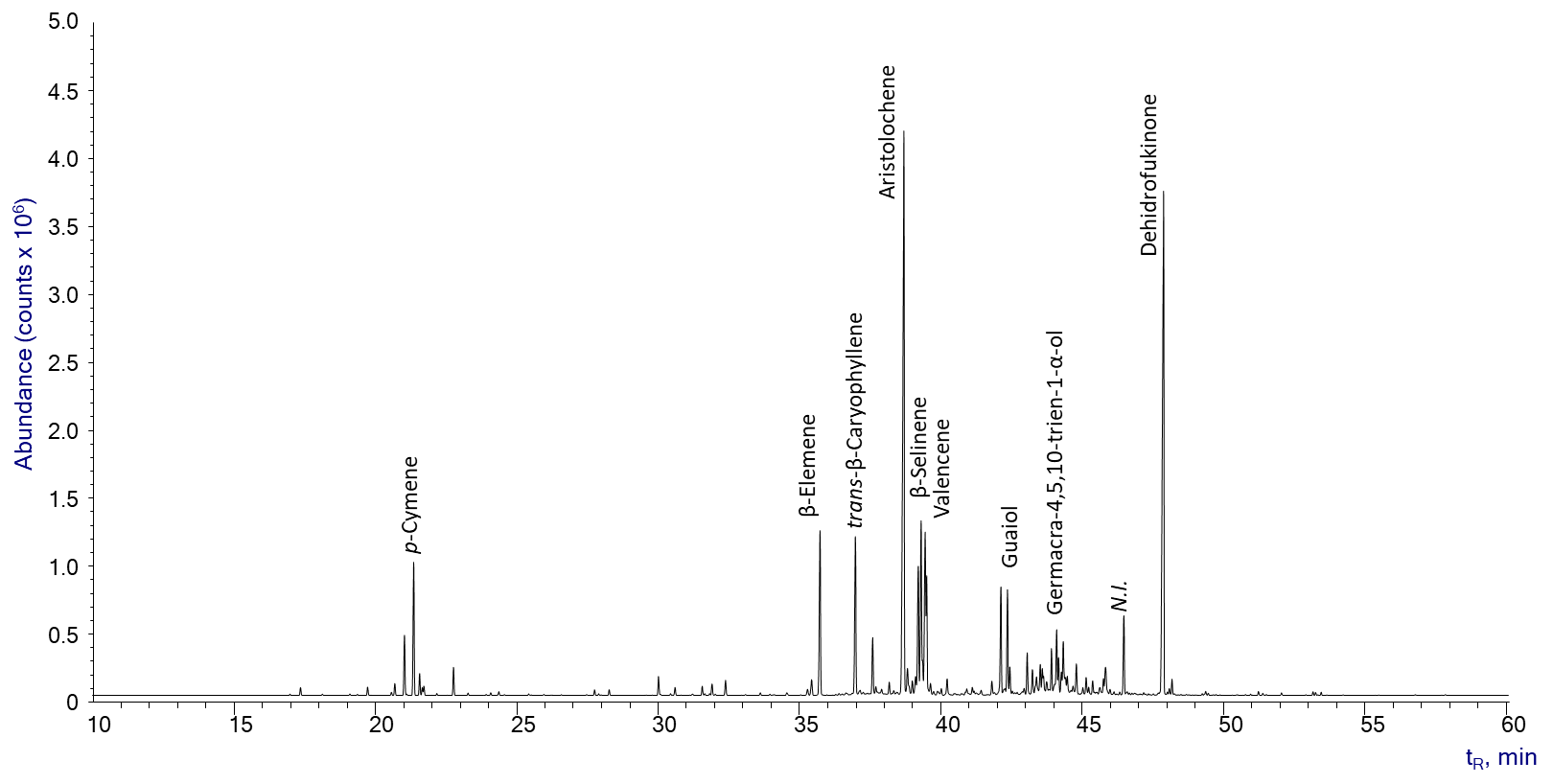


**S. Figure 12.** GC/MS of *Satureja viminea* EO code 0012


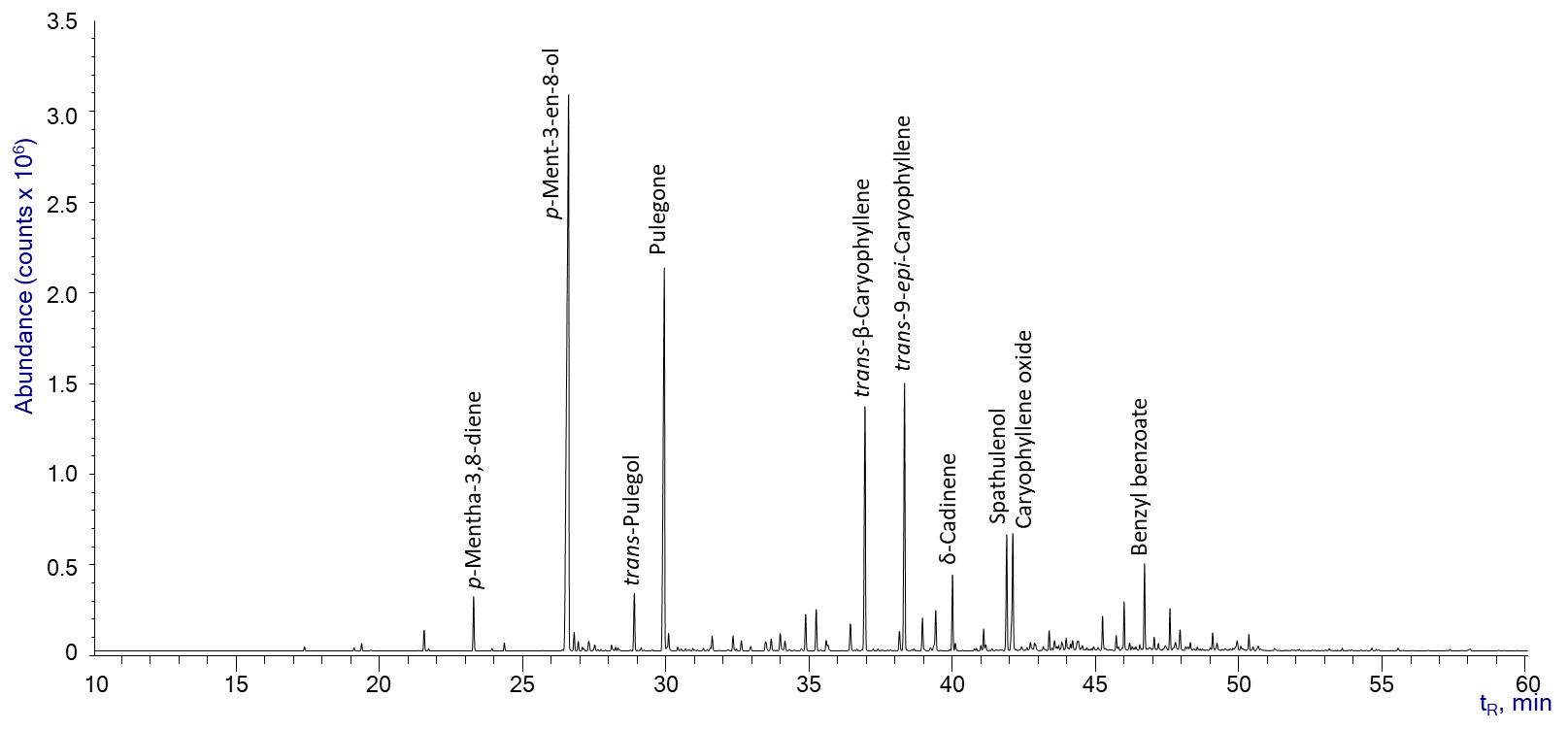


**S. Figure 14.** GC/MS of *Varronia curassavica* EO code 0014


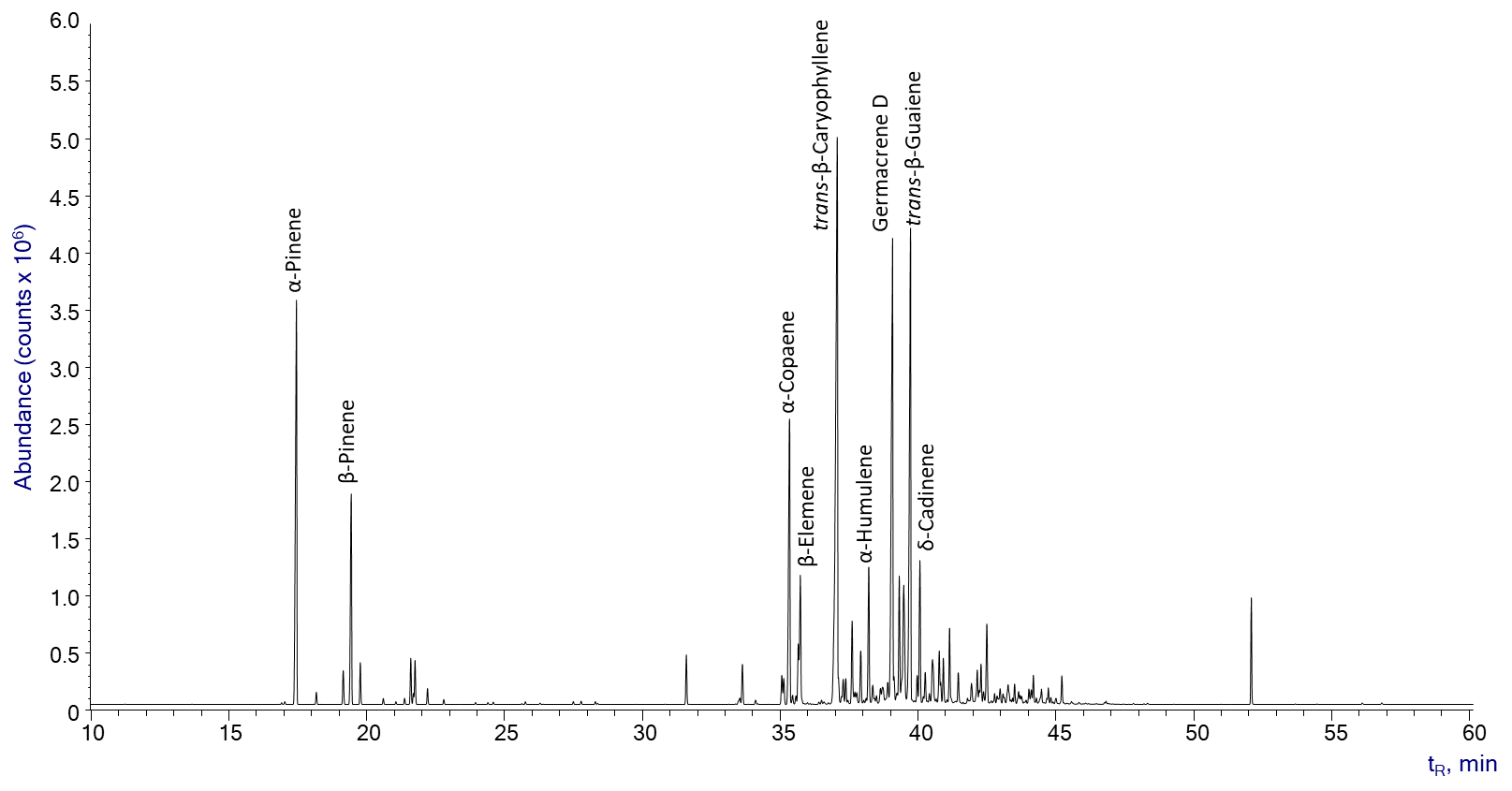


**S. Figure 15.** GC/MS of *Ocimum basilicum* EO code 0015


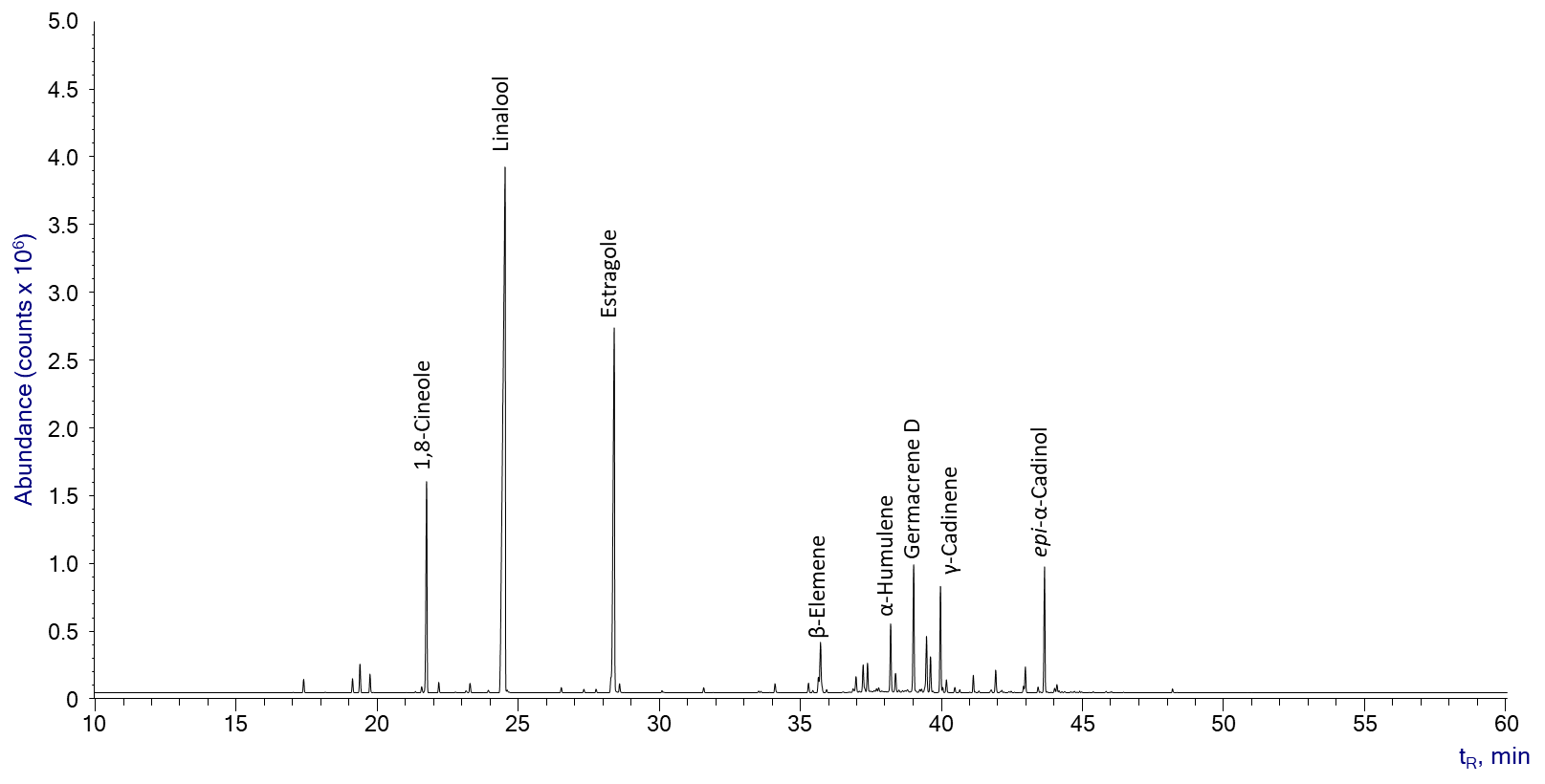


**S. Figure 16.** GC/MS of *Calycolpus moritzianus* EO code 0016


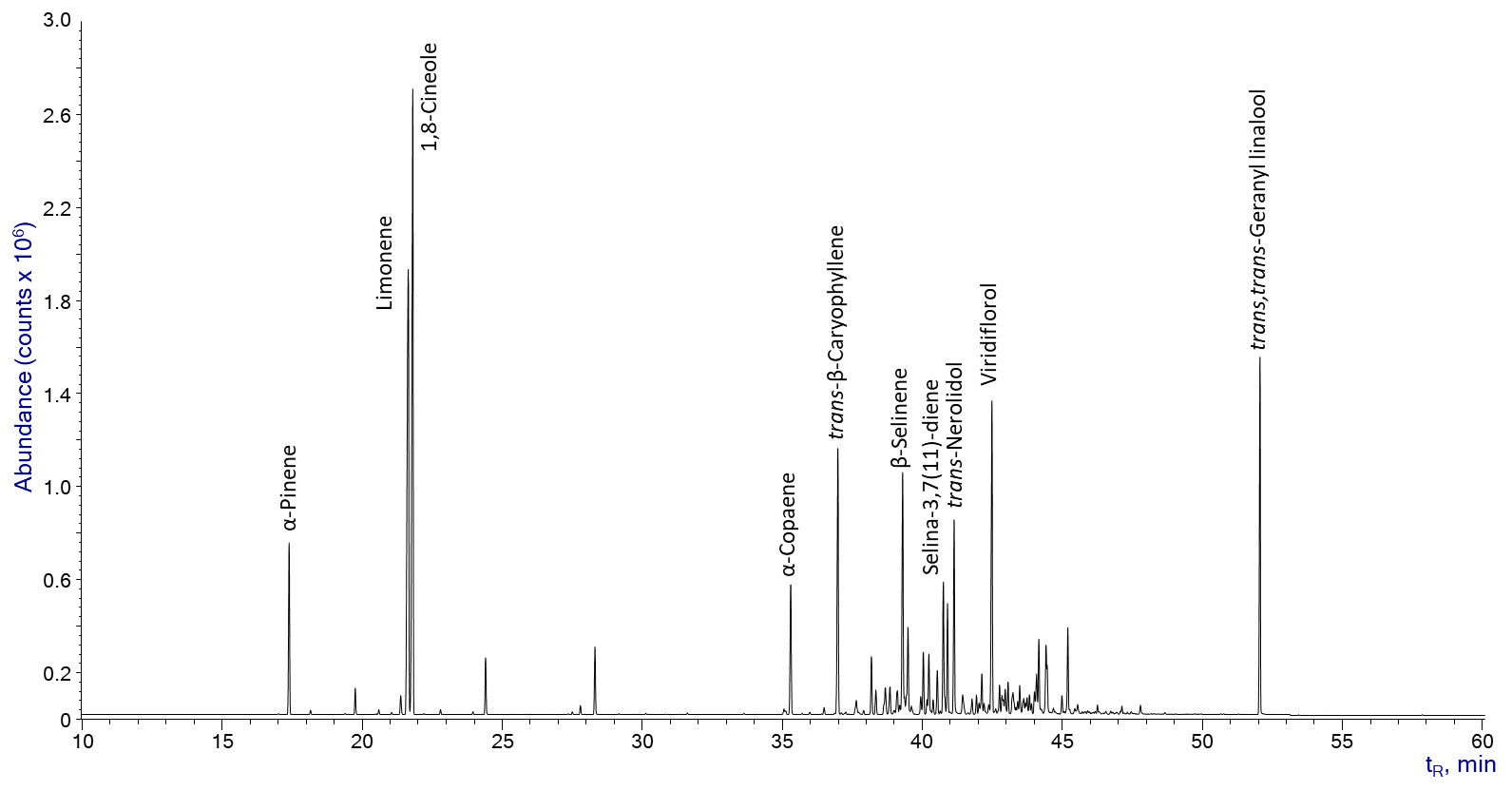


**S. Figure 17.** GC/MS of *Turnera diffusa* EO code 0017


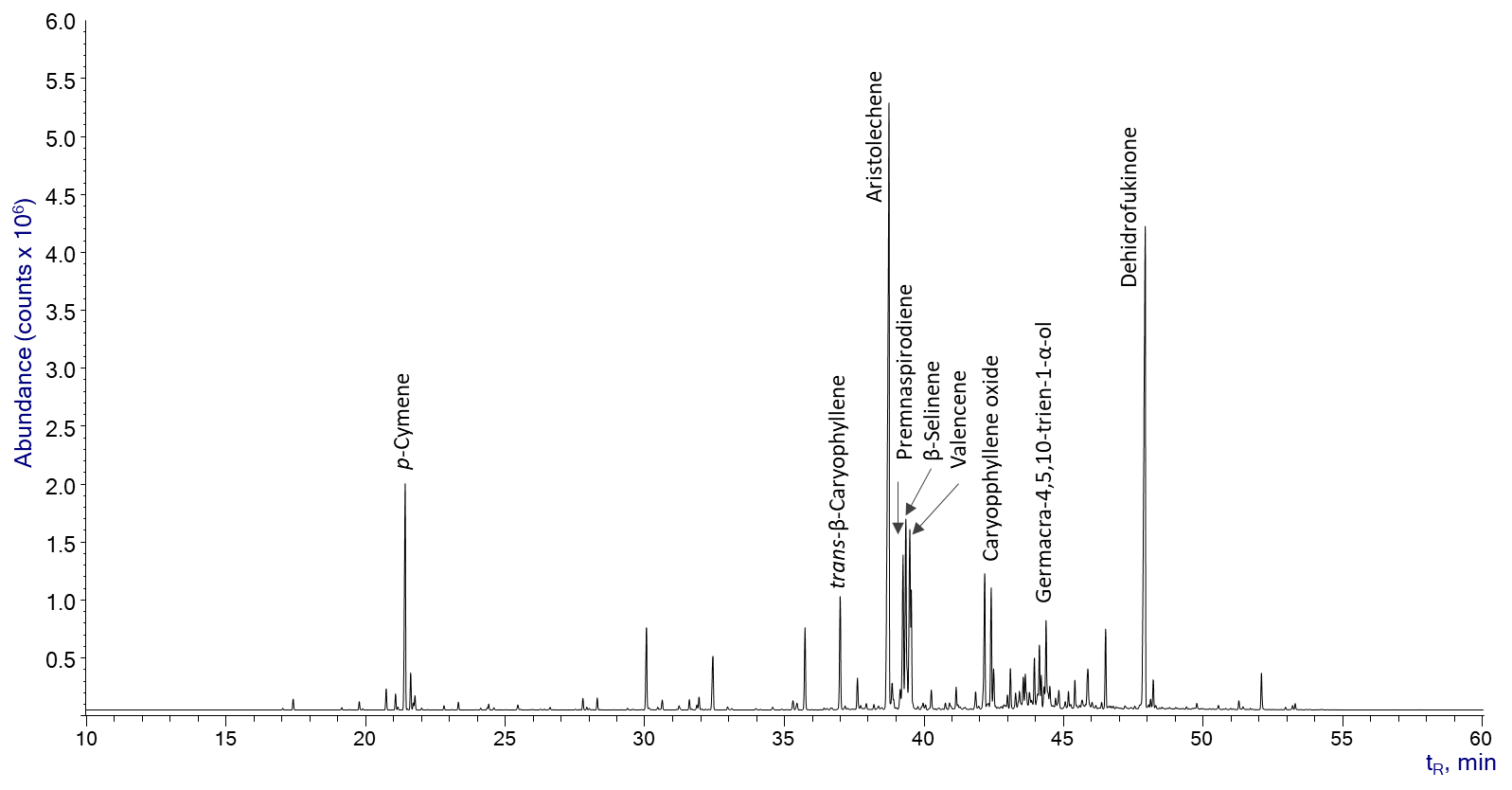


**S. Figure 18.** GC/MS of *Lippia origanoides chemotype thymol/p-cymene* EO code 0018


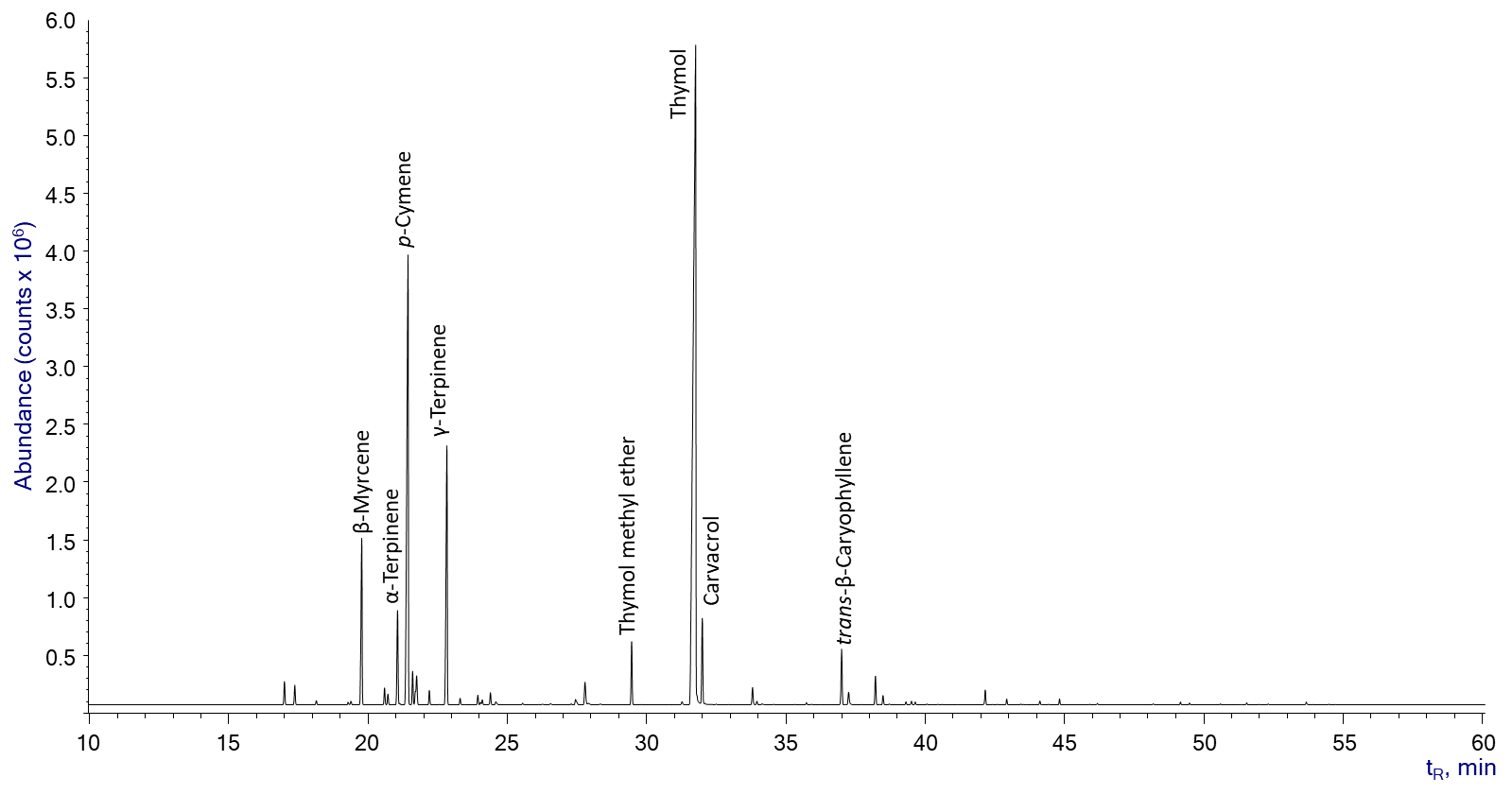


**S. Figure 19.** GC/MS of *Lippia origanoides chemotype thymol* EO code 0019


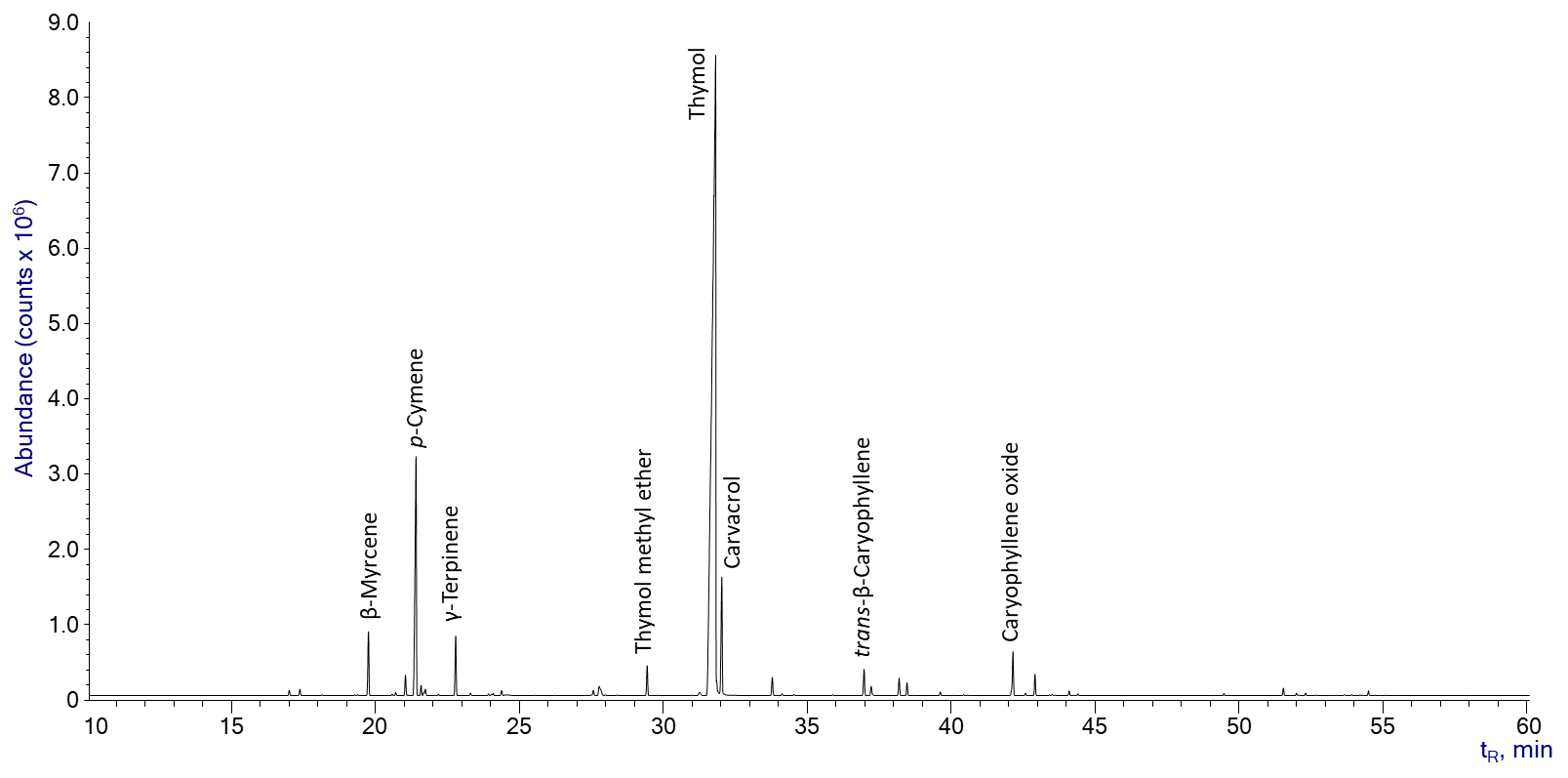


**S. Figure 20.** GC/MS of *Lippia micromera* EO code 0020


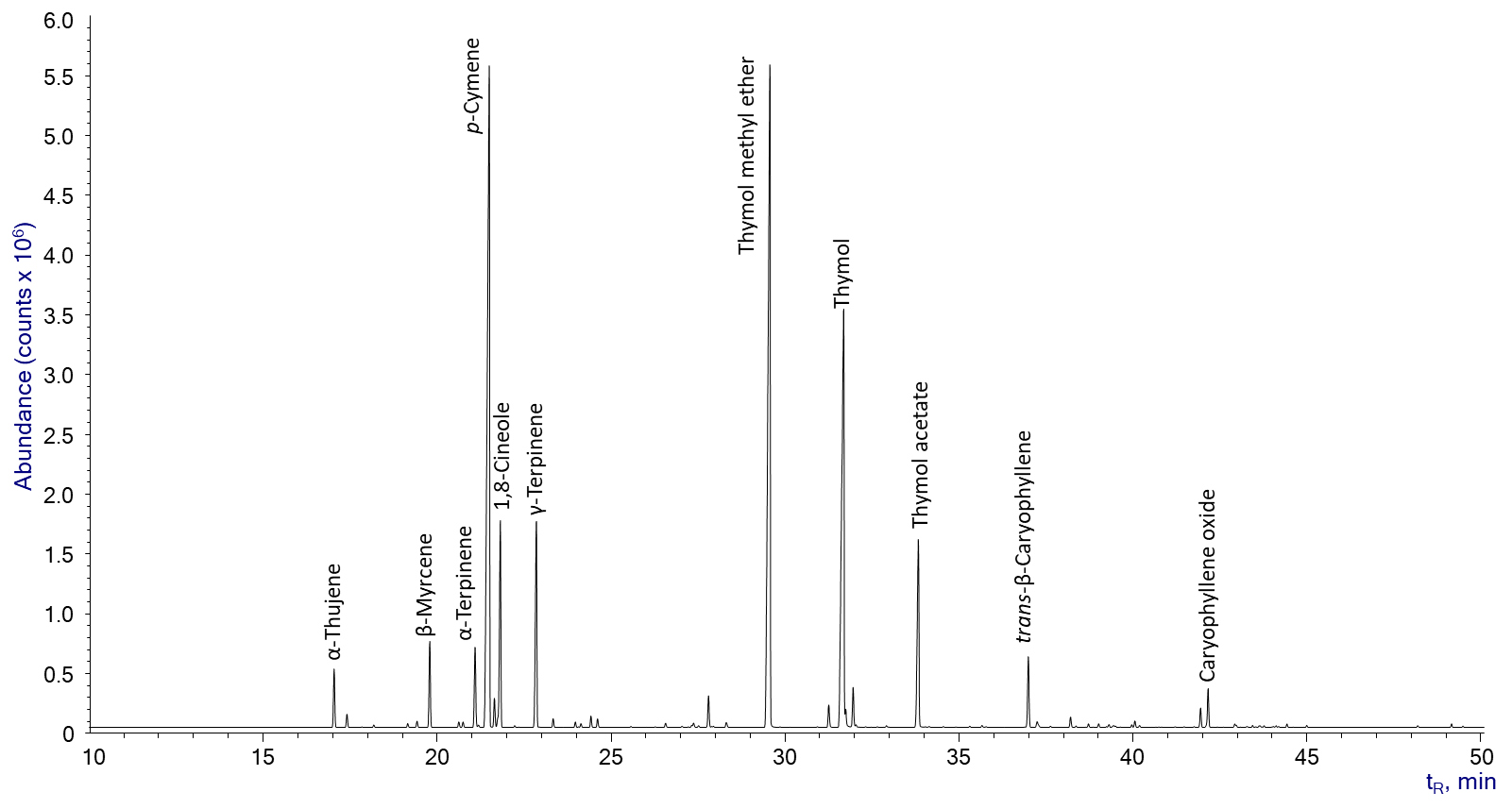


**
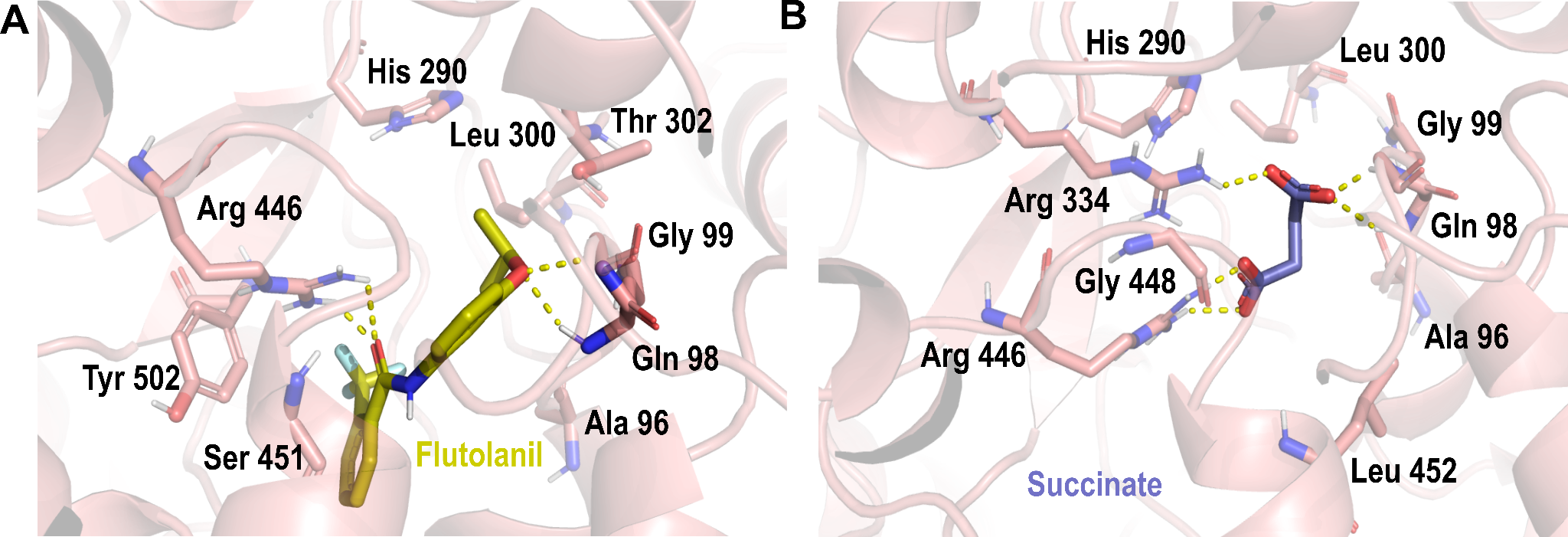
**

**S. Figure 21.** Docking poses of reference inhibitors of mitochondrial complex II in *Aedes aegypti*. **(A)** flutolanil; **(B)** succinate . Mitochondrial complex II’ residues are colored according to the atom type of the interacting amino-acid residues (protein’s carbon, salmon; oxygen, red; nitrogen, blue). The protein-ligand interactions are represented by dash lines as follows: hydrogen bond interactions are colored in yellow.


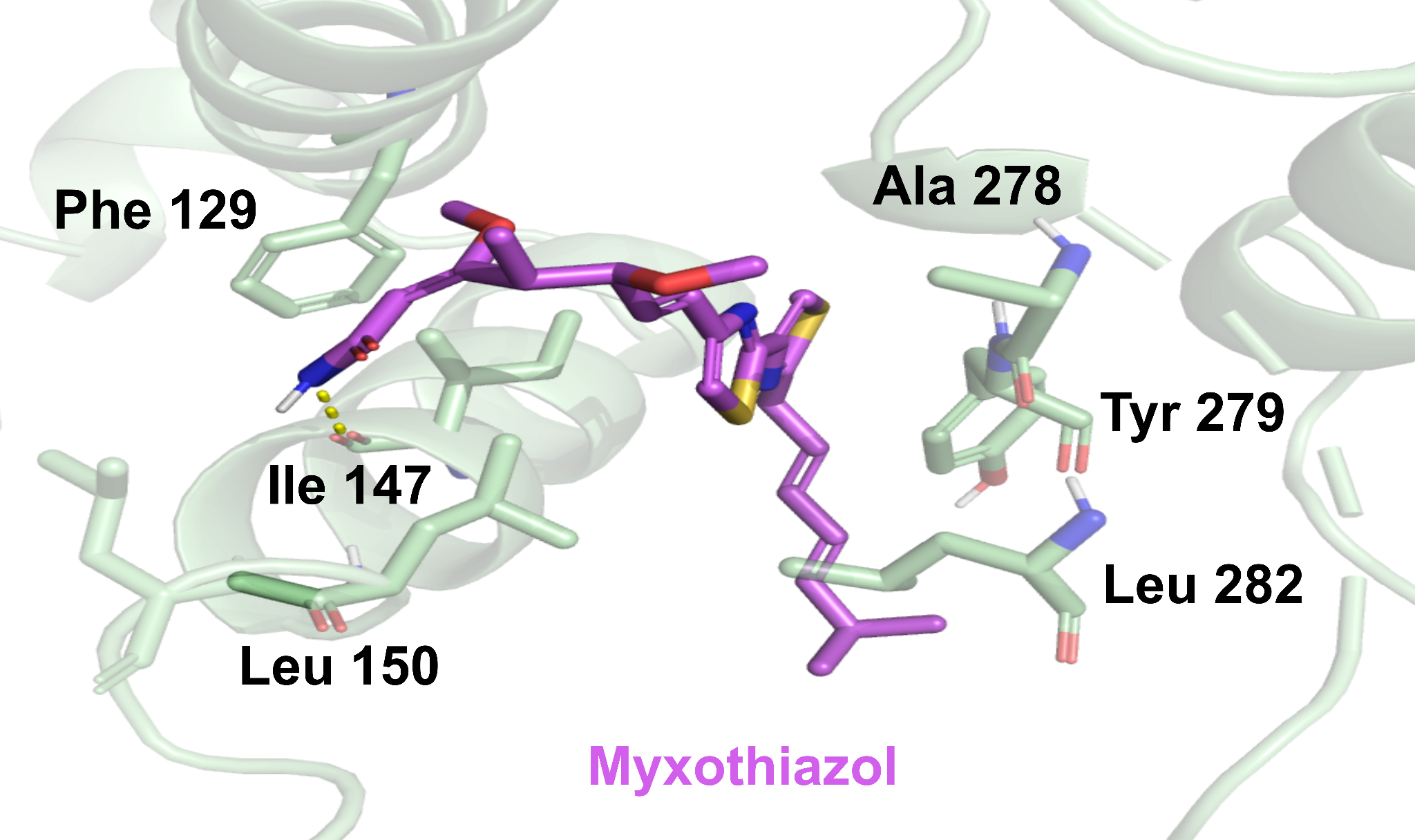


**S. Figure 22.** Docking pose of reference compound myxothiazol on subunit A of mitochondrial complex III in *Aedes aegypti*. Mitochondrial complex III’ residues are colored according to the atom type of the interacting amino-acid residues (protein’s carbon, pale green; oxygen, red; nitrogen, blue). The protein-ligand interactions are represented by dash lines as follows: hydrogen bond interactions are colored in yellow.


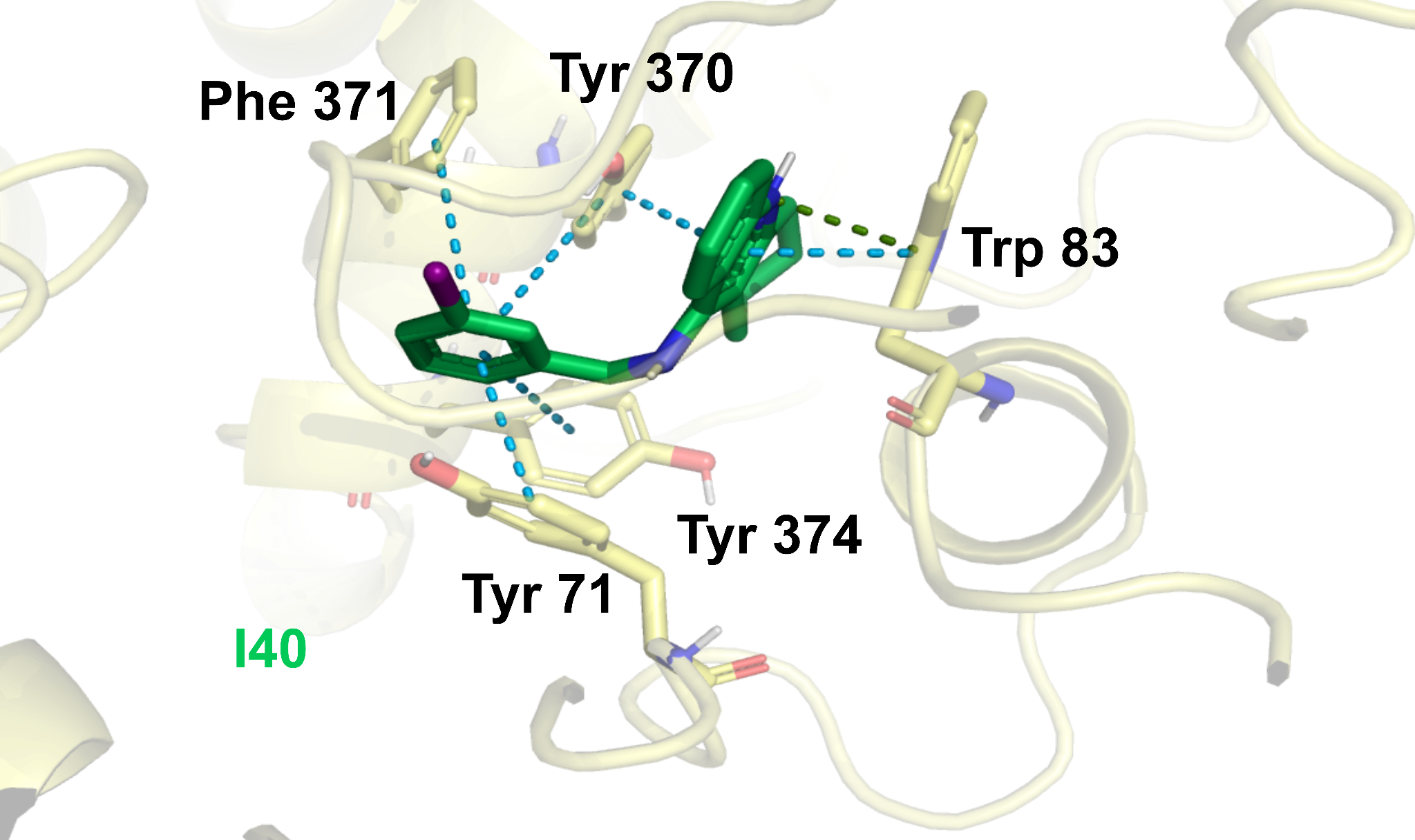


**S. Figure 23.** I40’s binding mode on the acetylcholinesterase crystal structure (PDBid: 1QON). Acetylcholinesterase' residues are colored according to the atom type of the interacting amino-acid residues (protein’s carbon, pale yellow; oxygen, red; nitrogen, blue). The protein-ligand interactions are represented by dash lines as follows: hydrogen bond interactions are colored in yellow, 𝜋-𝜋 interaction, and 𝜋-cation interactions are colored in blue and green, respectively.
